# Desymmetrization of Diboron(4) by a Trifluorination B-Masking Strategy: Practical Synthesis of Unsymmetrical Diboron Species

**DOI:** 10.1021/acs.joc.4c00715

**Published:** 2024-08-01

**Authors:** Nadim Eghbarieh, Ahmad Masarwa

**Affiliations:** Institute of Chemistry, The Center for Nanoscience and Nanotechnology, and Casali Center for Applied Chemistry, The Hebrew University of Jerusalem, Jerusalem 9190401, Israel

## Abstract

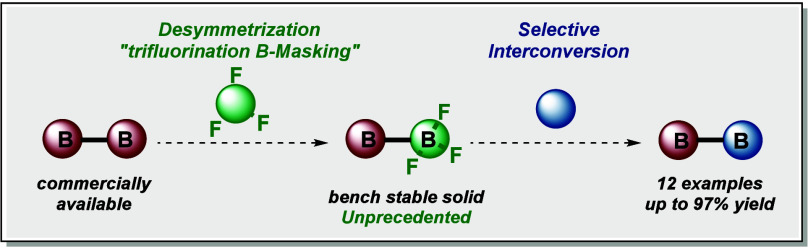

Herein, we report a straightforward practical and simple method for efficiently synthesizing a BF_3_-containing unsymmetrical diboron salt. This method involves the direct desymmetrization of commercially available diboron(4). This desymmetrization is based on a selective B-masking strategy via nucleophilic trifluorination, providing the elusive diborons bearing a trifluoroborate group. The synthetic utility of these salts is demonstrated through various examples of (sequential) B-ligand interconversions, enabling the synthesis of unsymmetrical diboron derivatives. These products, which serve as valuable building blocks, are obtained in gram-scale and high yields.

Although diboron species, with a boron–boron single bond, have been recognized for approximately a century, their chemistry has undergone thorough explorations only in the last three decades.^1,2^ These investigations revealed crucial structural features and reaction patterns, ultimately proving highly versatile and valuable in diverse synthetic pathways. These reagents, including the commercially available bis(pinacolato)diboron (B_2_pin_2_), have become instrumental in the synthesis of a wide array of valuable natural products, pharmaceutical intermediates, and biologically active compounds.^1^

The categorization of these diboron compounds can rely on the ligands surrounding the boron atoms.^1,2^ This involves considering not only the hybridization on the boron atom, such as neutral (sp^2^–sp^2^) or anionic (sp^2^–sp^3^), but also the symmetry of the diboron species, distinguishing between symmetrical and unsymmetrical configurations of these diborons. The inherent classification of these species, potentially leading to the polarization of the B–B bond, plays a crucial role in shaping their properties and reactivities. It also contributes significantly to determining the outcomes of regio- and stereoselectivities in their reactions.^1,2^

For example, the anionic (sp^2^–sp^3^) type species are among the more common reagents that are used in synthesis (Figure 1).^1−3^ This sp^2^–sp^3^ variety is often considered as a potential intermediate in many of the borylation reactions to construct selective organoboron compounds.^1,4^

**Figure 1:**
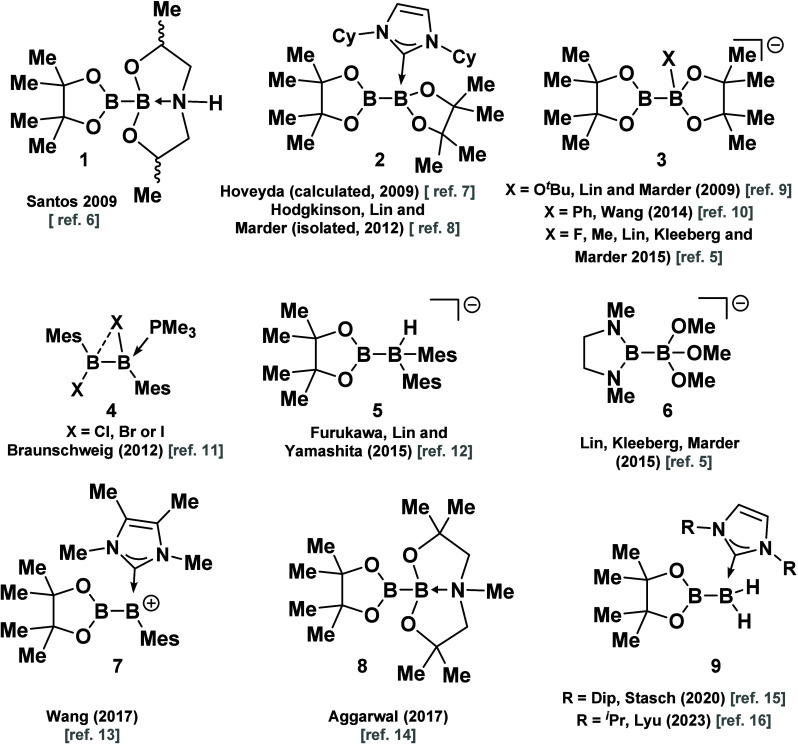
Selected examples of art prior works for the synthesis of unsymmetrical diboron sp^2^–sp^3^.

Therefore, it is important to investigate additional methods for synthesizing new variants of (unsymmetrical) diboron species and their synthetic applications. Such investigations are expected to greatly enhance the existing approaches for derivatizing noble diboron motifs and enable the creation of new connections. While the initial instance of sp^2^–sp^3^ hybridized diborane, i.e., monoetherate tetrachlorodiborane(4) (B_2_Cl_4_OR), was documented in 1949 by Schlesinger, the first conclusive confirmation of such a species occurred in 1972 by Timms and colleagues.^1,2^ In their work, the product (SiCl_3_)_2_B(CO)B(Cl)_2_ was identified as a neutral sp^2^–sp^3^ diborane, and the conformation of this species was corroborated by the observation of two signals–peaks in ^11^B NMR.^1,2^ Subsequently, in recent years, new methods for unsymmetrical diboron(4) synthesis have been developed that enable the creation of novel families of these species.^1−4^ Selected examples (**1**–**9**) of these methods are outlined in Figure 1.^5−16^

As part of our overarching strategies to synthesize a diverse range of unsymmetrical diboron based compounds (**11**–**14**), encompassing mixed groups of B(pinanediolato), Bpin, Bdan, Bmida, B(pin-*d*_12_), B(hex), B(neop), and other derivatives, we recognized the potential of anionic diboron (sp^2^–sp^3^) bearing a monotrifluoroborate salt group (e.g., Bpin-BF_3_M, **11a**) as a versatile core scaffold for these molecules (Figure 2). However, the synthesis and utilization of Bpin-BF_3_M salts have remained relatively obscure, and no reports, to the best of our knowledge, address their exploration (Figure 2).^1,2,17,18^ Therefore, it is imperative that a new general, mild, and diversifiable method for Bpin-BF_3_M salts be developed.^18^

**Figure 2:**
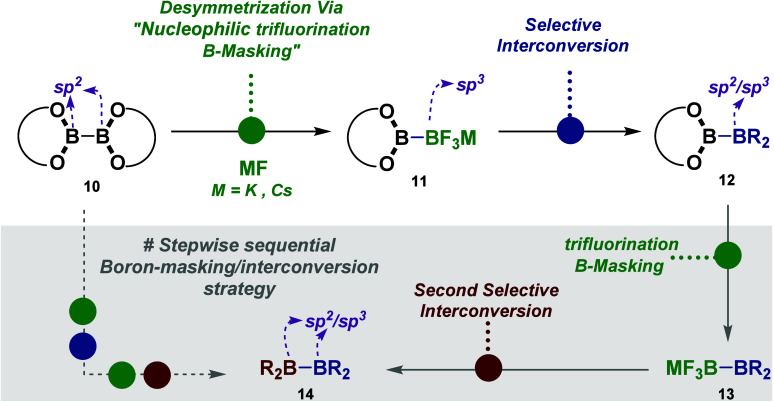
Overview of this work.

With this goal in mind, we envisioned an operationally simple strategy to synthesize Bpin-BF_3_M salts **11a** and convert them into valuable unsymmetrical diboron motifs (**12**) through the trifluoroborate (BF_3_) moiety transformation.^19^ In fact, drawing inspiration from our recent reports of B-masking protocols of polyborylated compounds,^20−23^ our method involves a simple desymmetrization of readily accessible and commercially available diboron starting materials by a nucleophilic trifluorination B-masking strategy leading to the proposed Bpin-BF_3_M salts. Yet, a key challenge in developing a B-masking strategy for these compounds (in **10**) lies in identifying a B-monoselective nucleophilic trifluorination reaction that specifically targets one boryl group of the B–B moieties, keeping the other boron position intact. However, activating this BF_3_ group provides the chance for conversion into other ligands.^19^ This activation also allows for the design of a sequential B-masking/interconversion approach, enabling the construction of novel unsymmetrical diboron derivatives as described in Figure 2.

To test our hypothesis and to address the challenge, we aimed to employ metal fluoride salts (MF) as a nucleophilic trifluorination source of nonetching conditions.^24^ Our investigation started with commercially available B_2_pin_2_ (**10a**) as the reactant to optimize the reaction conditions. After an extensive survey of the reaction parameters (for details, see Tables 1 and S1), the optimized conditions were identified as CsF (4 equiv), l-tartaric acid (2.5 equiv) as an alkali metal sponge (AMS),^24^ and a mixture of the solvents H_2_O, THF, MeOH, and CH_3_CN. We were pleased to observe **11a** as the sole product after only a 4 min reaction time, under green and mild conditions. The product was obtained as a solid by precipitation, thus making product isolation rapid and simple.

**Table 1:**
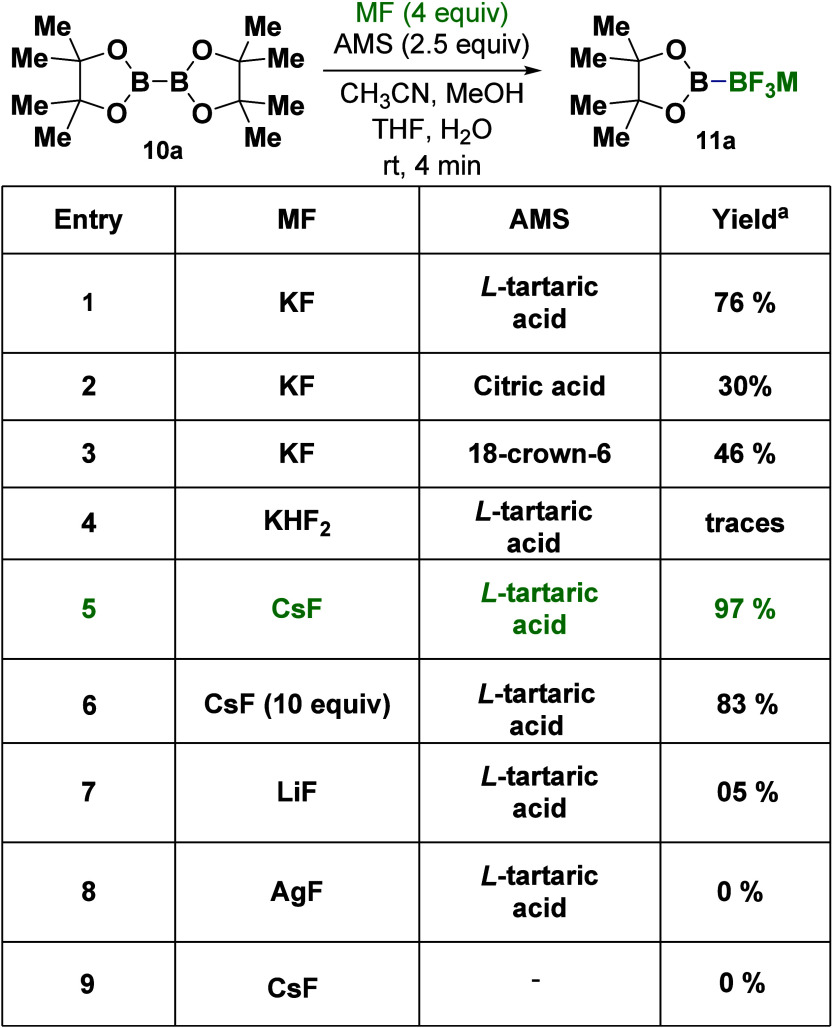
Trifluorination B-Masking Strategy for the Synthesis of Trifluorodiboron Salt **11a**^a^

a Reaction conditions: diboron **10a** (1 mmol) was dissolved in a mixture of 5 mL of CH_3_CN and 5 mL of MeOH. Subsequently, fluoride salt FM (4 mmol in 0.5 mL of H_2_O) and AMS (2.05 mmol in 3 mL of THF) were added in that order. For more details, see the Supporting Information.

Notably, product **11a** was not obtained in the presence of KHF_2_ or in the absence of AMS (entries 4 and 9, Table 1). Moreover, the selectivity for the production of **11a** was not affected when an excess of MF (entry 6) was used; in this case the yield slightly decreased to 83% (see Supporting Information). The reaction can be carried out on a gram scale (see **11a**, 19.7 mmol, 77%, Figure 3B).

**Figure 3:**
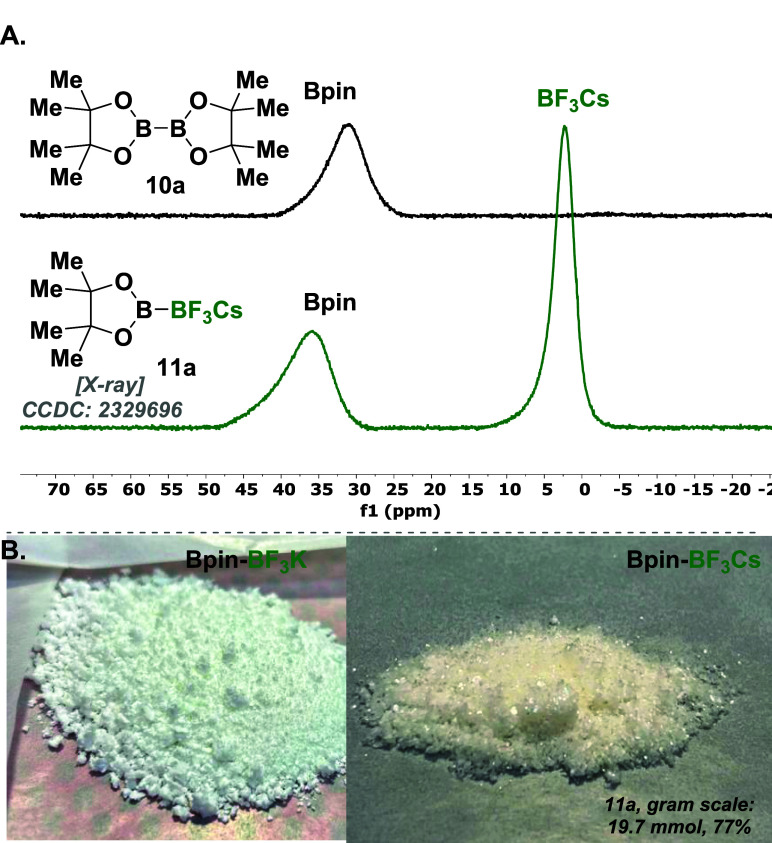
(A) ^11^B NMR comparison between substrate **10a** and product **11a**. (B) Pictures of product **11a** using KF (left side, Bpin-BF_3_K) and CsF (right side, Bpin-BF_3_Cs). It is noteworthy to highlight the distinct solid textures of both salts of **11a**, Bpin-BF_3_K and Bpin-BF_3_Cs. The former, with potassium (K), exhibits a powdery consistency, while the latter, with cesium (Cs), displays a more crystalline texture.

The ^11^B NMR spectra distinctly reveal two separate peaks: one at 35 ppm corresponding to the Bpin group and another at 4 ppm corresponding to the trifluoroborate BF_3_ group. This observation is evident in the comparison of the ^11^B NMR spectra of the starting material and the product **11a** in Figure 3A. The monoselectivity and the structure of product **11a** was unambiguously determined by X-ray crystallographic analysis (Figures 3A and 4B; see Table S2).

**Figure 4:**
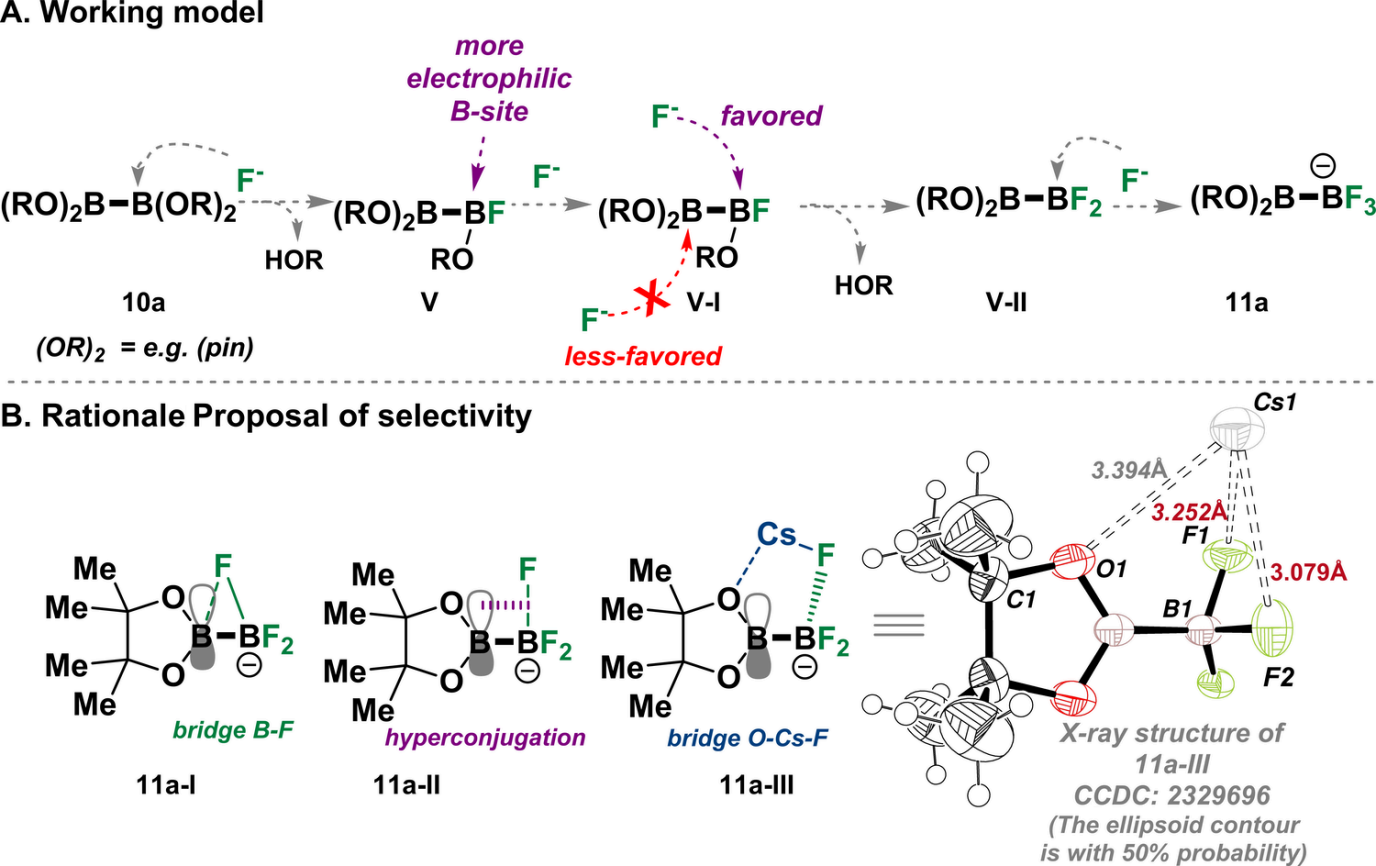
Proposed mechanism and rationales for the selective trifluorination B-masking strategy.

The same scenario was also successfully applied to the commercially available chiral bis[(−)pinanediolato]diboron **10b**, yielding the corresponding trifluoroborate product, chiral diboron B(pinanediolato)-BF_3_Cs **11b**, with a 42% yield (Scheme 1).

**Scheme 1:**
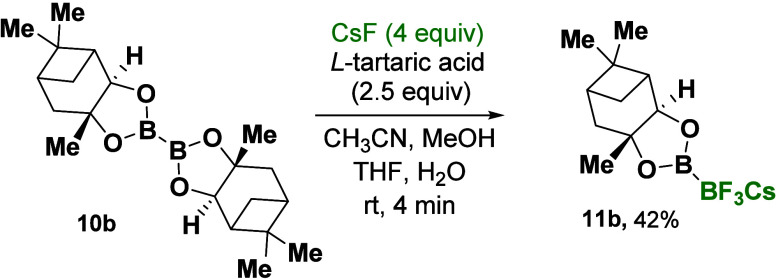
Trifluorination B-Masking Strategy for the Synthesis of Trifluorodiboron Salts (**11b**) Reaction conditions: Diboron **10b** (1 mmol) was dissolved in a mixture of 5 mL of CH_3_CN and 5 mL of MeOH. Subsequently, the fluoride salt CsF (4 mmol in 0.5 mL of H_2_O) and l-tartaric acid (2.05 mmol in 3 mL of THF) were added in that order. For more details, see the Supporting Information.

We believe that the mechanism underlying the selectivity, that is, the observation of only the mono-BF_3_Cs product while the other boron substituent remains untouched, arises from a “tandem” process in the fluorination step in which the first nucleophilic fluoride attacks the vacant *p*-orbital of one of the boron centers of **10a** (Figure 4A).^21,22^ As a result, this fluorinated boron (**V**, Figure 4A) becomes more electrophilic; hence, it forces the second and then the third nucleophilic fluorides to attack only the originally fluorinated boron center (**from V to 11a**, Figure 4A).^21,22^

Subsequently, the inquiry arises as to why the other boron substituent remains unaffected and does not undergo nucleophilic fluorination, even in the presence of an excess of CsF (10 equiv). In addressing this question, we propose three possible rationales.^21,22^ After the trifluorination event is completed, the generated trifluoroborate (BF_3_) moiety in **11a** develops a partial negative charge on the fluorides and this partial negative charge stabilizes the conjugated Bpin group against subsequent attack by fluoride; this stabilization may occur through a bridging fluoride structure (see **11a-I**, Figure 4B).^4,5,11,20−23^ Alternatively, the vacant p-orbitals on the boron atom (of the Bpin group in **11a**) may gain added stability through conjugation interaction with the sigma B–F bond, as described by **11a-II**.^4,5^ In this proposed scenario of **11a-II**, the boron is now shielded from nucleophilic attack (Figure 4B).

Finally, the X-ray structure revealed an intriguing rationale for an anticipated stabilizing interaction in the solid state of compound **11a** that might prevent the Bpin from undergoing nucleophilic fluorine attack (see the X-ray structure in Figure 4B). In this structure, the cesium counterion participates in chelation of between two of the fluorine atoms (Cs1–F1 3.252 Å, Cs1–F2 3.079 Å) and an oxygen (Cs1–O1 3.394 Å) from the pinacol moiety, resulting in the formation of a five-membered-ring-like bridge (**11a-III**, Figure 4B).^5^ It is noteworthy that these potential rationales (**11a-I**, **11a-II**, and **11a-III**) may concurrently contribute to the observed selectivity.

With these valuable diboron-BF_3_Cs (**11**) compounds in hand, we sought to demonstrate their synthetic utility in the selective transformations into diboron mixed boryl groups by interconversion of the BF_3_Cs group to new boryl-BR_2_ moieties as described in Scheme 2A.^19,25^ We were intrigued to observe that diboron-BF_3_Cs (**11**) could be selectively converted, for the first time, into mixed diboron containing a boronic ester moiety (**12**). Gratifyingly, the diboron-BF_3_Cs (**11**) can be directly converted to different unsymmetrical diborons (Scheme 2). The optimized conditions for converting **11a** (Bpin-BF_3_Cs) were determined utilizing potassium carbonate as a base, trimethylsilyl chloride, and the suitable diol or diamine in DMSO at room temperature for 3 h. This led to the formation of mixed diboron product **12** with good to excellent yields. Of note, trimethylsilyl chloride TMS-Cl was employed as the fluorophile.^19,25^ Notably, this approach facilitated the synthesis of the new diboron compound Bpin-B(hex) **12a** with a yield of 76% as well as the known Bpin-Bdan **12b** with a yield of 91%.^28^ Interestingly, employing pinacol-*d*_12_ as the diol resulted in a yield of 89% for the diboron product (Bpin-B(pin-*d*_12_) **12c**. Potentially, this deuterium-labeled diboron **12c** can be utilized in the mechanistic study for several reactions. Additionally, the diboron (sp^2^–sp^3^) product **12d**, Bpin-Bmida,^26^ was isolated with a yield of 67%. Furthermore, this method successfully converted **11b** into the new chiral B(pinanediolato)-Bdan (**12e**) with a yield of 77%. In addition, the scalability of this method was successfully demonstrated by the syntheses of **12b** (5 g, 80% yield) and **12d** (4 g, 60% yield, Scheme 2).

**Scheme 2:**
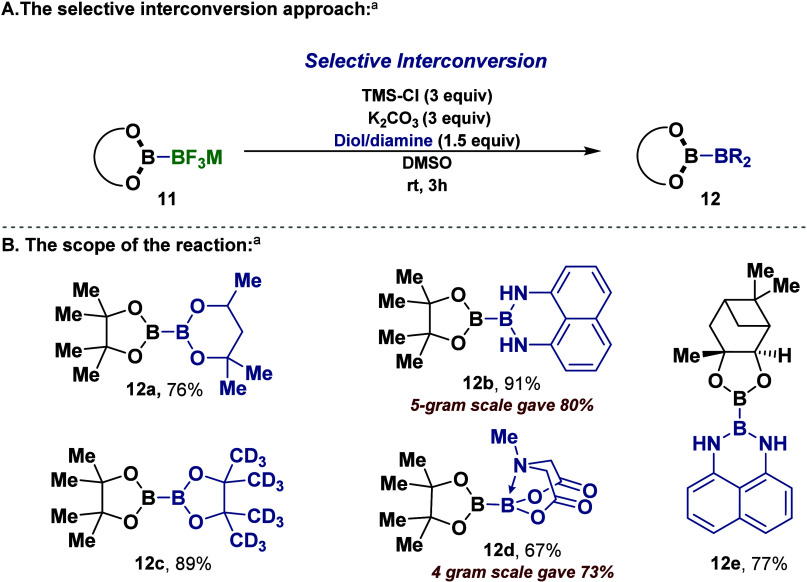
Selective (Sequential) Interconversion Reactions of (**11**) into Unsymmetrical Diborons (**12**) Reaction conditions: **11** (0.5 mmol, 1 equiv), K_2_CO_3_ (1.5 mmol, 3 equiv) and diol/diamine (0.75 mmol, 1.5 equiv) were dissolved in dry DMSO (6 mL) under nitrogen (N_2_) atmosphere. Then, TMS-Cl (1.5 mmol, 3 equiv) was added dropwise to the reaction. The reaction mixture was then stirred for 3 h at room temperature. Yields of isolated products are reported.

Next, we designed a programmed strategy outlining sequential selective B-masking/interconversion pathways for the synthesis of unsymmetrical diborons (Figure 5).^20−23^ Accordingly, we were able to develop this sequential masking protocol for the simple B_2_pin_2_**10a** affording the unsymmetrical diborons **13**–**14** (Figure 5).^26−28^ In this protocol, compound **10a** underwent selective fluorolysis creating Bpin-BF_3_Cs **11a** in high yield. Then **11** was selectively converted to Bpin-Bdan **12b**. Finally, **12b** underwent a trifluorination reaction of the Bpin moiety with retention of the Bdan to afford product Bdan-BF_3_Cs **13a**, in overall yield of about 83% from **10a**. Subsequent treatment of **13a** with TMS-Cl in the presence of different diols (pinacol-*d*_12_; hexylene-glycol; 2,2-dimethyl-1,3-propanediol) or diamine (1,8-diamino-naphthalene) gave the corresponding diborons B(pin-*d*_12_)-Bdan **14a**, B(hex)-Bdan **14b**, B(neop)-Bdan **14d**,^28^ and Bdan–Bdan **14c**,^27^ respectively (Figure 5). The products of these successive B-masking/conversions have been clearly determined by ^11^B NMR as described in Figure 5. Moreover, products **12b**, **13a**, and **14b** were prepared in a gram scale as shown in Figure 5. It is worth noting that these unsymmetrical diborons, specifically **11**, **12a**, **12c**, **12e**, **13a**, **14a**, and **14b**, belong to a family of compounds that currently face challenges in terms of efficient synthetic access.^1,2,17,27,28^

**Figure 5:**
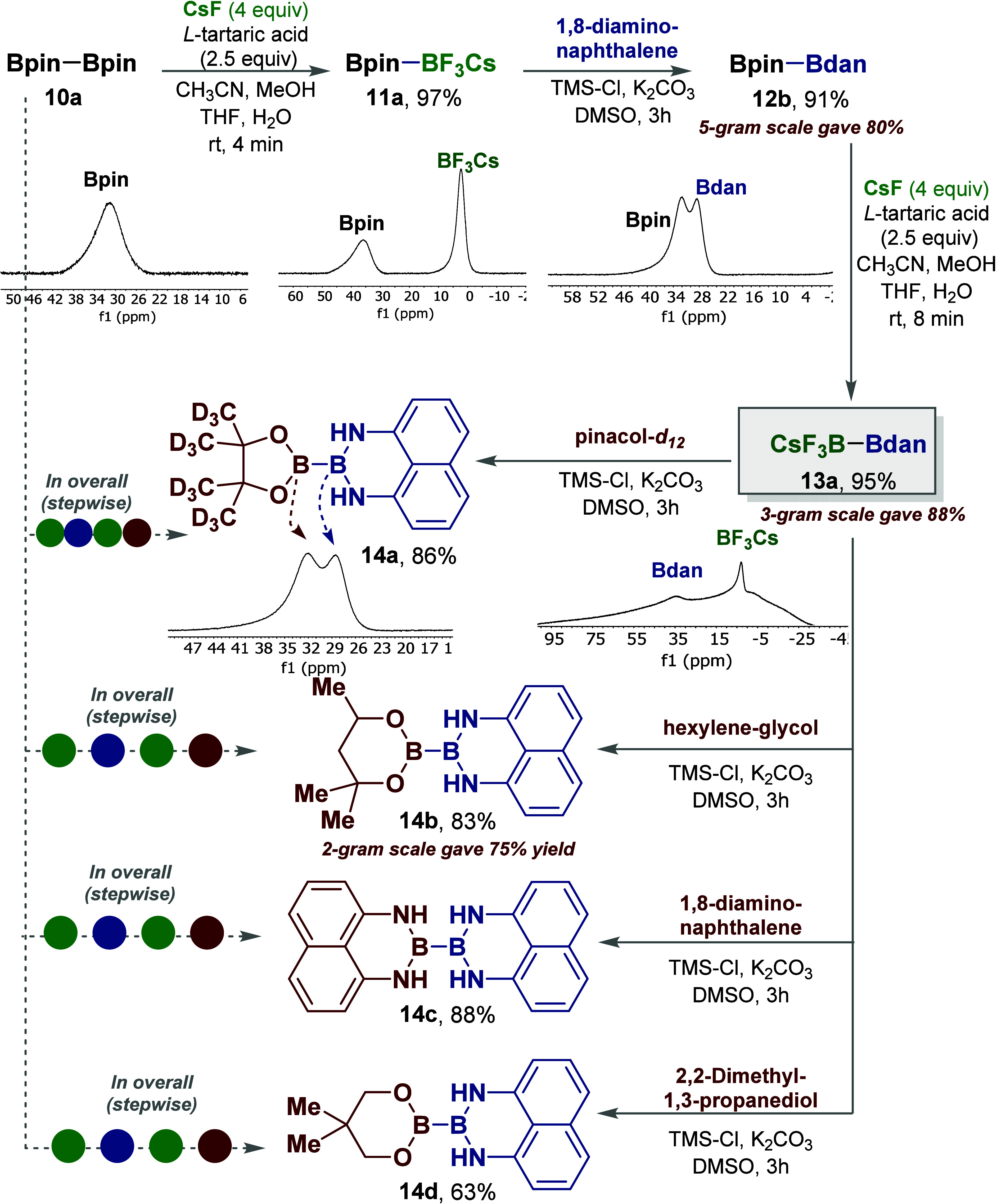
Stepwise sequential selective B-masking/interconversion for the preparation of unsymetrical diboron.

In conclusion, we have developed a novel masking strategy achieved through the initial desymmetrization of diboron(4) compounds employing nucleophilic trifluorination. This approach facilitates the transformation of symmetrical diboron structures into unsymmetrical diborons. The developed protocol proves to be a rapid, scalable, mild, versatile, and convenient method for synthesizing diverse substituted diborons featuring a trifluoroborate moiety. Notably, the process demonstrates high selectivity, particularly in the desymmetrization of commercially available diborons like B_2_pin_2_ and bis[(−)pinanediolato]diboron. The trifluoroborate group within compound **11** can be selectively converted into a range of unsymmetrical diboron species, highlighting the utility of **11** as an intermediate in a general method for interconverting diboron(4). Additionally, a selective sequential masking/interconversion strategy is outlined, utilizing simple setups and mild conditions. The resulting unsymmetrical diboron products, many of which serve as valuable building blocks, are obtained in high yields with excellent selectivities. These protocols are expected to significantly streamline the synthesis of unsymmetrical diboron-related compounds, further advancing their applicability in various synthetic endeavors.

## Experimental Section

### Representative General Procedure for the Synthesis of Compound **11a**

The reaction took place in an open-air environment. Diborone (**10**; 1 mmol, 1 equiv, 254 mg) was dissolved in a mixture of acetonitrile (5 mL) and methanol (5 mL) in an open flask. To this mixture, a solution of a fluoride salt [4 mmol, 4 equiv, either KF (232.3 mg in 0.5 mL of H_2_O) or CsF (604 mg in 0.5 mL of H_2_O)] was added, and the resulting mixture was stirred at room temperature for 1 min. Then, l-tartaric acid (2.05 mmol, 2.05 equiv, 307 mg in 3 mL of THF) was added dropwise to the rapidly stirred turbid solution. During this addition, a white precipitate formed. The reaction mixture was filtered to remove the white precipitate and washed thoroughly with excess acetonitrile (10 mL). The filtrate was then concentrated in a rotary evaporator to obtain a crude solid. Subsequent washing with diethyl ether and hexane yielded the corresponding trifluoroborate (**11a**) as a white solid, which was further dried under high vacuum overnight.

#### 4,4,5,5-Tetramethyl-2-(trifluoro-l4-boraneyl)-1,3,2-dioxaborolane, Cesium Salt (**11a**)

White solid (317 mg, 97% yield), mp = 305–307 °C. A 5 g reaction gave product **11a** in (4.986 g, 77% yield). ^1^H NMR (400 MHz, DMSO-*d*_6_) δ 1.08 (s, 12H); ^13^C{^1^H} NMR (101 MHz, DMSO-*d*_6_) δ 80.1, 25.1; ^11^B NMR (128 MHz, DMSO-*d*_6_) δ 36.1, 2.3; ^19^F NMR (376 MHz, DMSO-*d*_6_) δ −26.7 (s); HRMS (Q-TOF) *m*/*z*: [M]^−^ Calcd for C_6_H_12_B_2_O_2_F_3_ 195.0983; Found 195.0984.

All details, general information, experimental procedures, characterization data, and NMR spectra for products in Table 1 and Schemes 1 and 2 and Figures 3–5 are gathered in the Supporting Information.

## Data Availability

The data underlying this study are available in the published article and its Supporting Information.

## Supporting Information Available

Chemical compound information, experimental procedures, product characterization data, X-ray data, NMR spectra (PDF)
