# Supplementary material for: Desymmetrization of Diboron(4) by a Trifluorination B-Masking Strategy: Practical Synthesis of Unsymmetrical Diboron Species
Source: J Org Chem. 2024 Aug 1;89(16):11753–60. doi: 10.1021/acs.joc.4c00715 (PMC11334178; doi:10.1021/acs.joc.4c00715)
Supplement: Supplementary file 1 — jo4c00715_si_001.pdf [file jo4c00715_si_001.pdf]

Supplementary Information

Desymmetrization of Diboron (4) by a Trifluorination B-Masking Strategy: Practical Synthesis of Unsymmetrical Diboron Species

Nadim Eghbarieh[1] and Ahmad Masarwa[1]*

[1]Institute of Chemistry, The Center for Nanoscience and Nanotechnology, Casali Center for Applied Chemistry, The Hebrew University of Jerusalem, Jerusalem 9190401 (Israel).

*Corresponding author: E-mail: Ahmad.Masarwa1@mail.huji.ac.il.

Table of Contents

1. Notes……………………………………………..…..……………………….………........S2

1.1. Materials and General Remarks……………………………………………………......S2

2. Methods……………………………………………………………......................................S3

2.1 Information and preparation for Starting Materials………………………………..S3

2.2 Procedure-A and Characterizations for Trifluorodiboron salt Products (**11**)……........S4

2.3 Procedure-B and Characterizations for Interconversion to Products (**12**)……..……S10

2.4 Procedure-C and Characterizations for Products (**13a**)…………………………..….S15

2.5 Procedure-D and Characterizations for Products (**14**)………………………...…......S17

3. X-Ray Crystallography Data …………………………………………………….….……….S20

4. NMR Spectra……….……………………………………..…………..…………….…….S21

5. References……………………………………..….…..……..............................….……S42

1. Notes.

1.1 Materials and General Remarks.

Unless otherwise stated, reactions were performed in oven-dried glassware fitted with rubber septa under inert atmosphere (N_2) and were stirred with teflon-coated magnetic stirring bars. Materials and chemicals were purchased from Sigma-Aldrich Inc., Combi-Blocks Inc., Alfa Aesar., and other commercial suppliers. Liquid reagents and solvents were transferred via syringe using standard Schlenk techniques. Solvents DMSO, hexane, methanol and acetonitrile were used as commercial grade and used as received with no further drying. Tetrahydrofuran (THF), diethyl ether (Et_2O), and dichloromethane (CH_2Cl_2) were used from a solvent purification system. All other reagents were used as received unless otherwise noted. Thin layer chromatography (TLC) was performed using silica gel 60 F-254 pre-coated plates (0.25 mm) and visualized by UV irradiation ƛ=232 nm, CAM stain, $KMnO_4$ stain, and other stains. Silica gel of particle size 230-400 mesh was used for flash chromatography, Flash chromatography (FC) was performed using CombiFlash, with SiO_2 columns. ^{1}H-NMR and ^{13}C-NMR spectra were recorded on 400, 500 MHz spectrometers with ^{13}C-NMR operating frequencies of 101, 126 MHz, ^{11}B-NMR operating frequencies of 128, 160 MHz, ^{19}F-NMR operating frequencies of 376, 471 MHz respectively. ^{1}H-NMR and ^{13}C-NMR spectra were referenced to TMS as an internal standard with a deuterated solvent unless otherwise stated. X-Ray structure was visualized with PLATON[1]. Chemical shifts (δ) are reported in ppm relative to the residual solvents ($CDCl_3$) signal (δ = 7.26 for ^{1}H-NMR and δ = 77.16 for ^{13}C-NMR) and (DMSO-d_6) signal (δ = 2.50 (ppm) for ^{1}H-NMR and δ = 39.5 (septet) for ^{13}C-NMR). Data for ^{1}H-NMR spectra are reported as follows: chemical shift (multiplicity, coupling constants, and number of hydrogen). Abbreviations are as follows: s (singlet), d (doublet), t (triplet), q (quartet), p (pentate), m (multiplet), brs (broad singlet). High-Resolution Mass Spectrometry (HRMS) were recorded on a SCIEX, X500$_R$ Q-TOF-MS (Quadrupole-TOF) using acetonitrile or methanol as solvent in a direct injection. Melting points (m.p) were determined with Stuart Scientific SMP 10 melting point apparatus.

2. Methods.

2.1. Information and preparation for Starting Materials.

All reagents were purchased from a commercial source and used as received unless otherwise noted. bis(pinacolato)diBoron (**10a**) (Apollo Scientifiv), bis[(-)pinanediolato]diBoron (**10b**) (Combi-Blocks), 2-methylpentane-2,4-diol (Sigma-Aldrich), 1,8-diaminonaphthalene (Combi-Blocks), methyliminodiacetic acid (Combi-Blocks) and 2,2-dimethyl-1,3-propanediol (Sigma-Aldrich), Cesium Fluoride (Combi-Blocks), Potassium Fluoride (Sigma-Aldrich), *L*-tartaric acid (Combi-Blocks), and Trimethylsilyl-Chloride (Sigma-Aldrich).

Pinacol-*d₁₂* [2,3-bis(methyl-d3)butane-1,1,1,4,4,4-d6-2,3-diol] was prepared in accordance to previous reported procedure.[2]

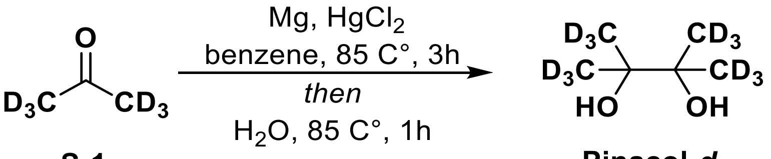

S-1 **Pinacol-*d₁₂***

Under nitrogen (N_2) atmosphere, magnesium (1 g, 68 mmol) was added into a three necked, flame dried round bottom flask, 20 mL of dry benzene was then added into the same flask and the mixture was stirred for 5 minutes at room temperature. Then a solution of mercury (II) chloride (1.84 g, 6.8 mmol) in acetone-d_6 (**S-1**) (68 mmol, 5 mL) was added dropwise through a dropping funnel and kept stirring until the vigorous reaction was complete. Then another 5 mL of acetone-d_6 (**S-1**) with benzene (10 mL) was added and reaction was refluxed using an oil bath for 3 hours. While hot, water (2.5 mL) was added carefully to the reaction mixture and the mixture was stirred for additional 1 hour at reflux. After that, the mixture was filtered while hot, the solids were returned to the flask and refluxed using an oil bath for 5 minutes with a fresh benzene (10 mL), and filtered again while hot. The filtrates were then combined, and reduced to one half of its volume using a rotary evaporator. Then water (5 mL) was added dropwise to the remaining clear solution of benzene while cold, resulting in the precipitation of pinacol-d_{12} hydrate as a white solid that was further washed with benzene. Pinacol-d_{12} hydrate was hydrated by heating the solids at 150 C$^{\circ}$ using an oil bath for 3 hours and then distilled to give 3.5 g (40 % yield) of the desired pinacol-d_{12} as a yellowish gummy solid. The spectral data are consistent with those reported in the literature.[2]
^{1}H NMR (400 MHZ, $CDCl_3$) δ 2.46 (s, 2H).

2.2. Procedure-A and Characterizations for Trifluorodiboron salt Products (11).

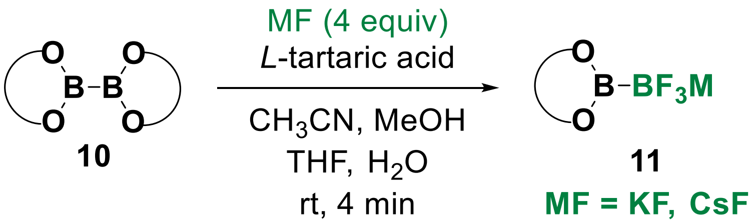

Procedure-A: The reaction took place in an open air environment. Diborone (**10**) (1 mmol, 1 equiv, 254 mg) was dissolved in a mixture of acetonitrile (5 mL) and methanol (5 mL) in an open flask. To this mixture, a solution of a fluoride salt [4 mmol, 4 equiv, either KF (232.3 mg in 0.5 mL H_2O) or CsF (604 mg in 0.5 mL H_2O)] was added, and the resulting mixture was stirred at room temperature for 1 minute. Then, *L*-tartaric acid (2.05 mmol, 2.05 equiv, 307 mg in 3 mL THF) was added dropwise to the rapidly stirred turbid solution. During this addition, a white precipitate formed. The reaction mixture was filtered to remove the white precipitate and washed thoroughly with excess acetonitrile (10 mL). The filtrate was then concentrated in a rotary evaporator to obtain a crude solid. Subsequent washing with diethyl ether and hexane yielded the corresponding trifluoroborate (**11**) as a white solid, which was further dried under high vacuum overnight.

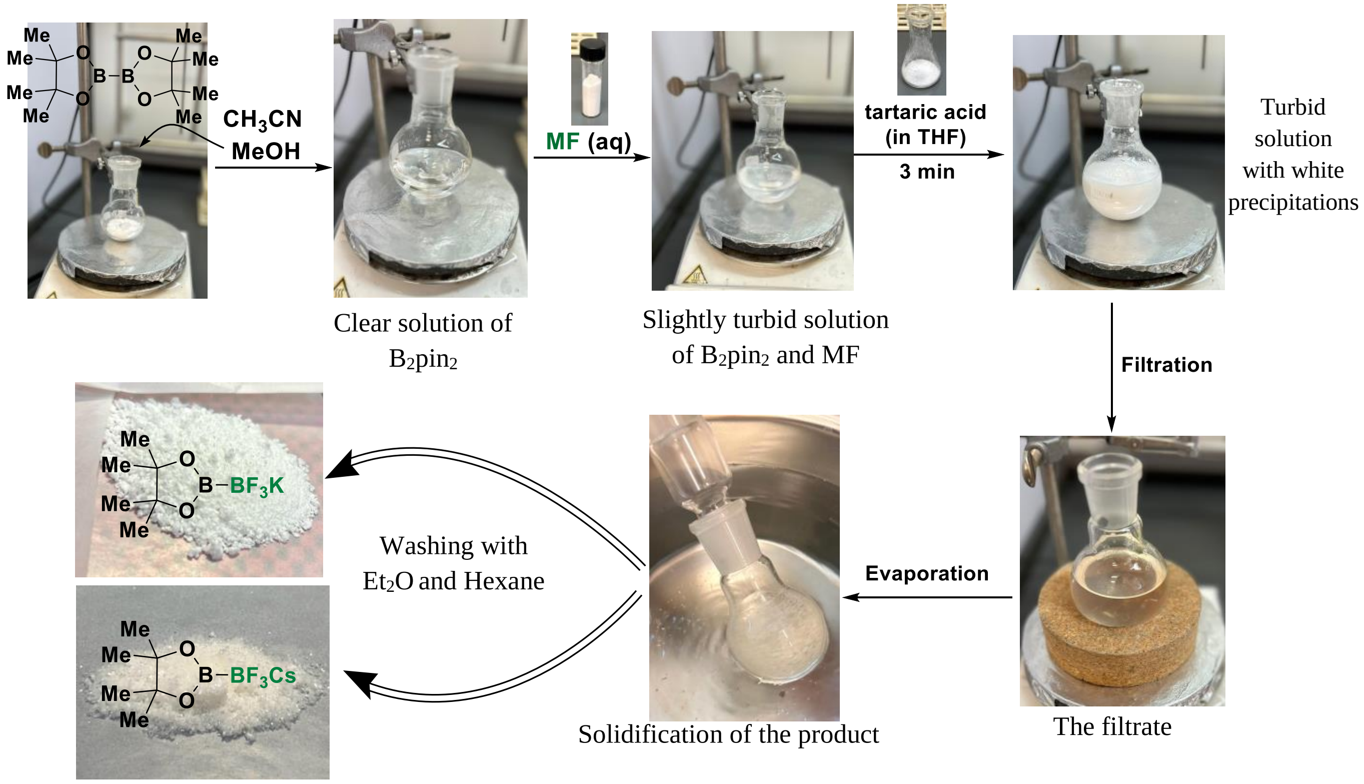

Figure S1: Reaction setup for the preparation of product **11a**.

Table S1. Optimization table for trifluoroborate salts (**11a**) preparation:

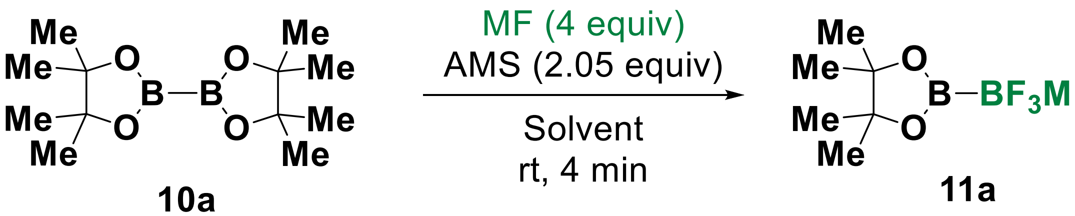

Entry	MF	Solvent 1	Solvent 2	Solvent 3	AMS	Time	Yield of (11a)[a,b]
1	KF	CH_3CN	MeOH	THF	*L*-tartaric acid	4 min	76 %
2	KF	CH_3CN	MeOH	THF	Citric acid	4 min	30 %
3	KF	CH_3CN	MeOH	THF	18-crown-6	4 min	46 %
4	KHF_2 (2 equiv)	-	MeOH	-	-	5 hours	traces
5	KHF_2	CH_3CN	MeOH	THF	*L*-tartaric acid	4 min	traces
6	KF	-	MeOH	THF	*L*-tartaric acid	4 min	10 %
7	CsF	CH_3CN	MeOH	THF	*L*-tartaric acid	4 min	97 %
8	CsF (10 equiv)	CH_3CN	MeOH	THF	*L*-tartaric acid (5 equiv)	4 min	83 %
9	CsF	CH_3CN	MeOH	THF	-	4 min	traces
10	CsF	CH_3CN	-	THF	*L*-tartaric acid	4 min	00 %
11	CsF	Acetone	MeOH	THF	*L*-tartaric acid	4 min	20 %
12	LiF	CH_3CN	MeOH	THF	*L*-tartaric acid	4 min	05 %
13	TBAF	CH_3CN	MeOH	THF	-	4 min	FC
14	AgF	CH_3CN	MeOH	THF	*L*-tartaric acid	4 min	00 %

[a] Reactions were carried out with 0.20 mmol of **10a** and 0.8 mmol of MF along with 2 mL of Solvent-1, 2 mL of Solvent-2, 0.7 mL of Solvent-3 and 0.41mmol of AMS (Alkali Metal Sponge) in an open flask at rt for the indicated amount of time. [b] Isolated yield. [c] full conversion (FC) by ^{1}H-NMR.

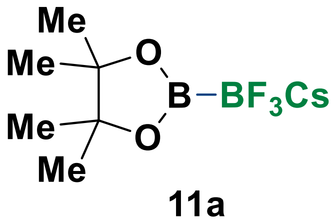

11a

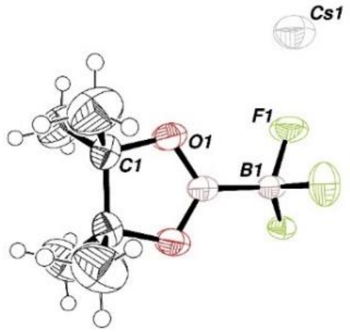

X-ray structure of 11a
CCDC : 2329696

The ellipsoid contour is with 50% probability level.

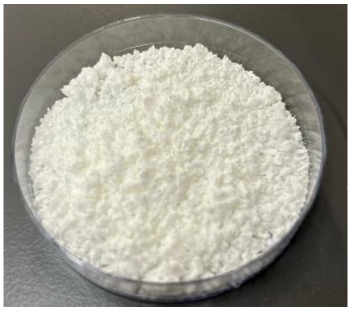

Figure S2: A picture of product **11a** from a large-scale synthesis.

4,4,5,5-tetramethyl-2-(trifluoro-l4-boraneyl)-1,3,2-dioxaborolane, cesium salt:

Prepared according to general Procedure-A using bis(pinacolato)diboron (B_2pin_2) (**10a**) (1 mmol, 358 mg), product (**11a**) was isolated in (317 mg, 97% yield) as a white crystal solid. A 5-gram reaction gave product **11a** in (4.986 gr, 77% yield).

mp: 305 - 307 °C.

^{1}H NMR (400 MHZ, DMSO-d_6) δ 1.08 (s, 12H).

^{13}C{^{1}H} NMR (101 MHz, DMSO-d_6) δ 80.1, 25.1.

^{11}B NMR (128 MHz, DMSO-d_6) δ 36.1, 2.3.

^{19}F NMR (376 MHz, DMSO-d_6) δ -126.7 (s).

HRMS (Q-TOF) m/z: $[M]^-$ Calcd for $C_6H_{12}B_2O_2F_3$ 195.0983; Found 195.0984.

Note: (1) The structure of **11a** was confirmed by X-ray crystallographic analysis, CCDC 2329696 (see supplementary table 2, page 15). (2) Compound **11a** was recrystallized by using acetone as a solvent (3) Using KF as a fluorine source formed (**Bpin-BF_3K**) in 76% yield as a white powder without any noticeable change to the ^{11}H, ^{13}C, ^{11}B, ^{19}F-NMR. Melting point showed a value of mp: 295 - 296.

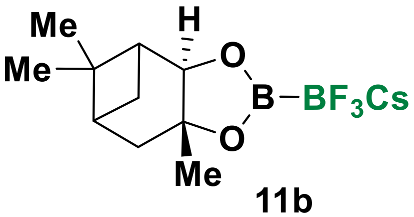

11b

(3aR,7aR)-5,5,7a-trimethyl-2-(trifluoro-l4-boranyl)hexahydro-4,6methanobenzo [1,3,2]dioxaborole, cesium salt:

Prepared according to general Procedure-A, using bis[(-)pinanediolato]diboron (**10b**) (1 mmol, 358 mg), product (**11b**) was isolated in (160 mg, 42% yield) as a white solid.

mp: 305 - 309 °C.

^{1}H NMR (400 MHZ, DMSO-d_6) δ: 3.98 (dd, J_1 = 8.6 Hz, J_2 = 1.8 Hz, 1H), 2.23 - 2.16 (m, 1H), 2.08 - 2.02 (m, 1H), 1.87 (t, J = 5.6 Hz, 1H), 1.82 - 1.78 (m, 1H), 1.71 - 1.59 (m, 1H), 1.22 (d, J = 8.5 Hz, 6H), 0.98 (d, J = 10.4 Hz, 1H), 0.81 (s, 3H).

^{13}C{^{1}H} NMR (101 MHz, DMSO-d_6) δ 82.8, 75.2, 50.9, 37.4, 35.2, 28.9, 27.0, 25.9, 23.7.

^{11}B NMR (128 MHz, DMSO-d_6) δ 34.4, 2.2.

^{19}F NMR (376 MHz, DMSO-d_6) δ -126.6 (s)

HRMS (Q-TOF) m/z: [M]$^-$ Calcd for $C_{10}H_{16}B_2O_2F_3$ 247.1298; Found 247.1292.

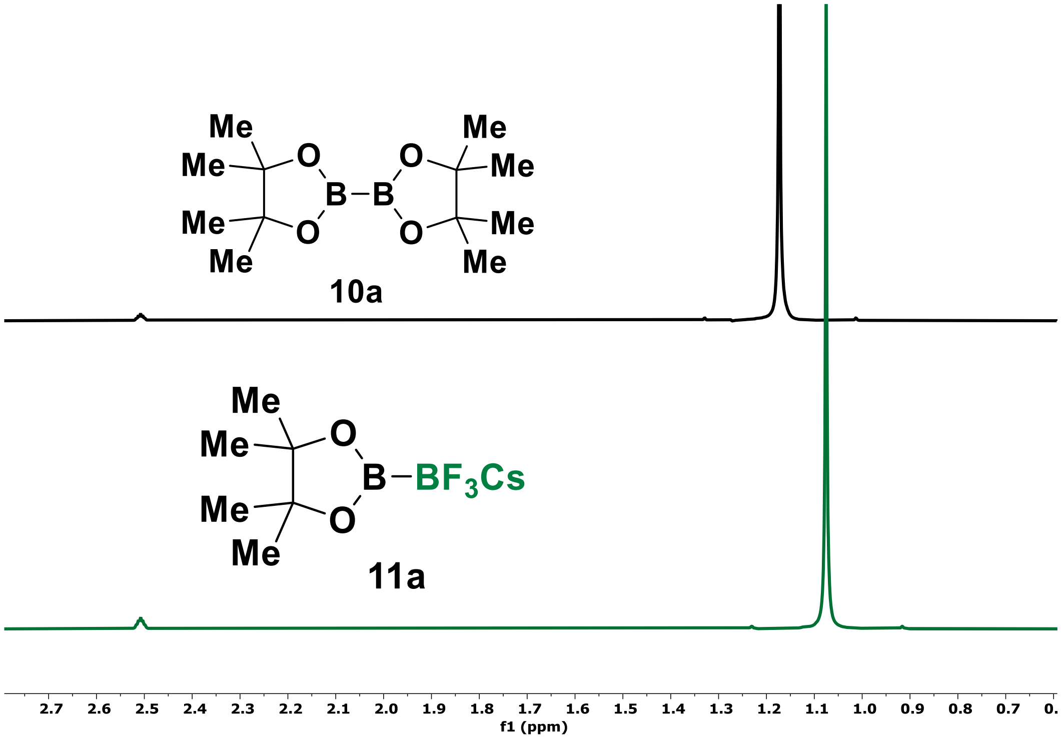

Figure S3: Comparison of ^{1}H-NMR for **10a** and **11a**.

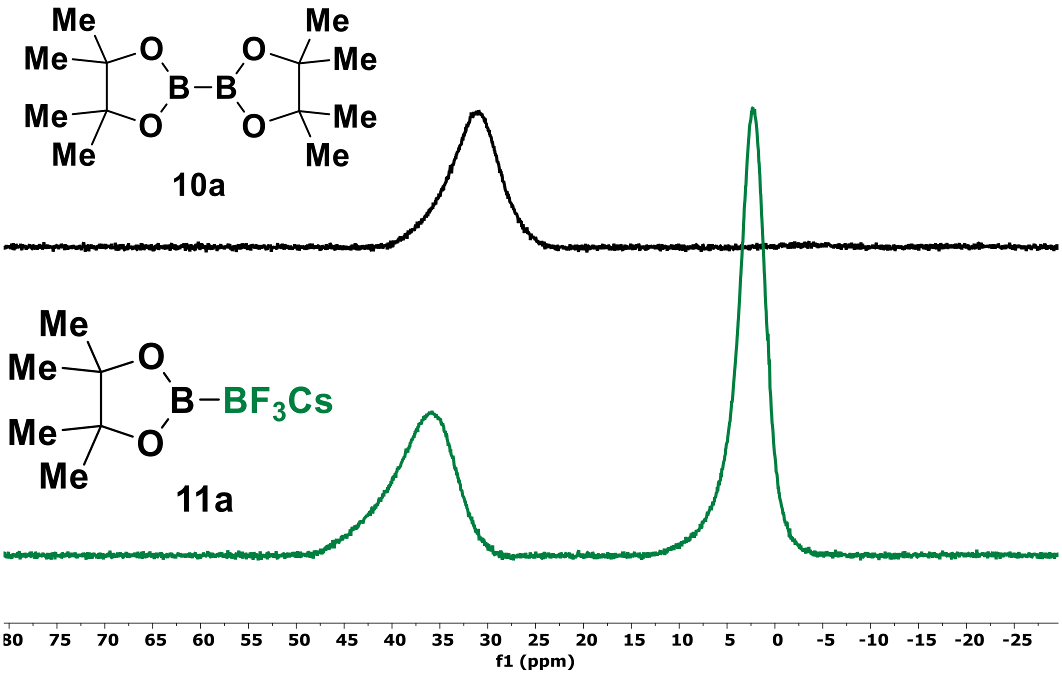

Figure S4: Comparison of ^{11}B-NMR for **10a** and **11a**.

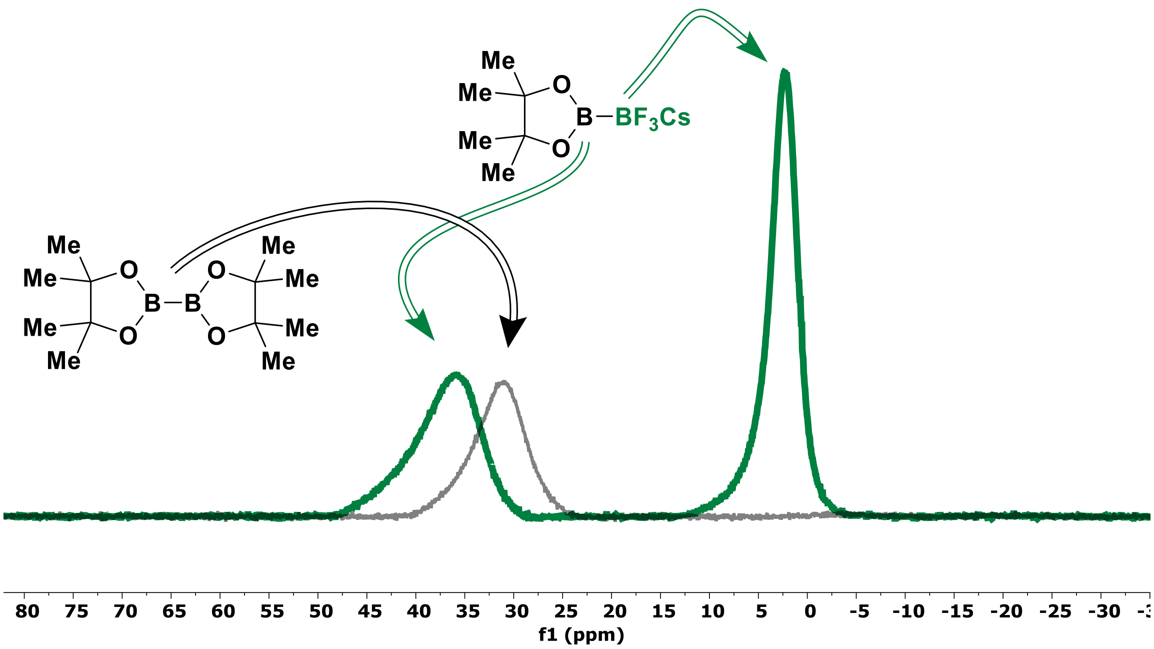

Figure S5: Comparison of ^{11}B-NMR for **10a** and **11a**.

2.3 Procedure-B and Characterizations for Interconversion to Products (12).

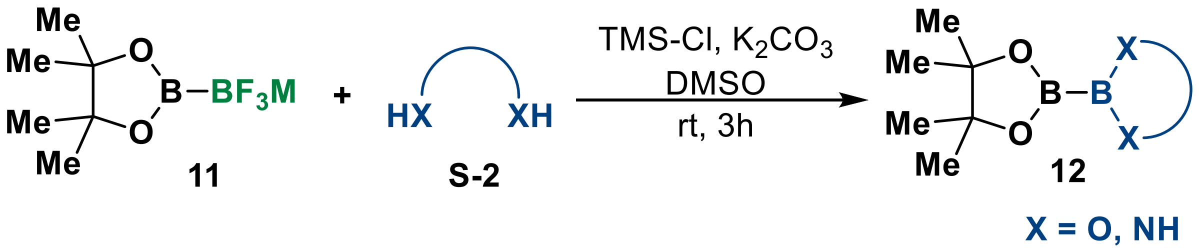

Procedure-B: Inside an oven-dried 25 mL Schleck tube, Bpin-BF_3Cs diborane (**11a**) (0.5 mmol, 1 equiv, 163 mg), K_2CO_3 (1.5 mmol, 3 equiv, 207 mg), and diol/diamine (**S-2**) (0.75 mmol, 1.5 equiv) were added and dissolved in dry-DMSO (6 mL) under nitrogen (N_2) protection. To the above mixture, trimethylsilyl chloride TMS-Cl (1.5 mmol, 3 equiv, 190 µL) was added dropwise, and the reaction was left stirring for 3h at room temperature. After completion of the reaction, EtOAc (10 ml) and H_2O (5 ml) were added, and the residue was extracted with EtOAc (3 × 8 mL). The organic layers were combined and washed with brine, dried over $MgSO_4$, and concentrated under reduced pressure using evaporator to obtain a crude material. The crude material was further purified by a short column on silica gel, resulting in the formation of product (**12**).

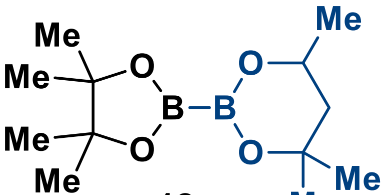

12a

4,4,6-trimethyl-2-(4,4,5,5-tetramethyl-1,3,2-dioxaborolan-2-yl)-1,3,2-dioxaborinane:

Prepared according to Procedure-B, using 2-methylpentane-2,4-diol (0.75 mmol, 88 mg, 1.5 equiv) as a diol. Product (**12a**) was isolated in (96.9 mg, 76 % yield) as a white solid by silica gel chromatography (EtOAc/Hexane = 15:85). R_f = 0.43 (15% EtOAc in Hexane).

mp: 110 - 112 °C.

^{1}H NMR (400 MHZ, $CDCl_3$) δ 4.16 (dqd, J_1 = 12.1 Hz, J_2 = 6.2 Hz, J_3 = 2.8 Hz, 1H), 1.76 (dd, J_1 = 13.9 Hz, J_2 = 3.0 Hz, 1H), 1.52 (dd, J_1 = 13.8 Hz, J_2 = 11.7 Hz, 1H), 1.32 - 1.21 (m, 21H).

^{13}C{^{1}H} NMR (101 MHz, $CDCl_3$) δ 83.1, 70.6, 64.4, 46.5, 31.2, 28.5, 25.1, 25.0, 23.2.

^{11}B NMR (128 MHz, $CDCl_3$) δ 29.6.

HRMS (Q-TOF) m/z: $[M+H]^+$ $C_{12}H_{25}B_2O_4$ Calcd for 255.1937; Found 255.1933.

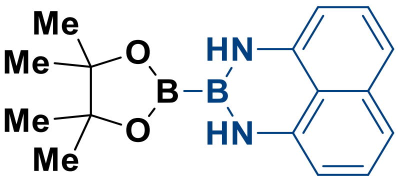

12b

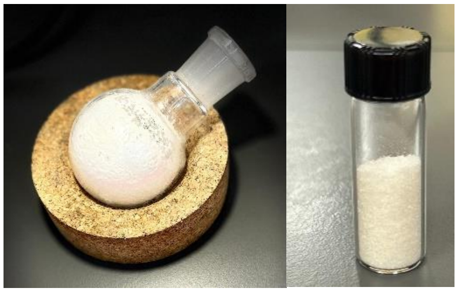

Figure S6. Pictures of product **12b** from a large-scale synthesis.

2-(4,4,5,5-tetramethyl-1,3,2-dioxaborolan-2-yl)-2,3-dihydro-1H-naphtho[1,8-de][1,3,2]diazaborinine:

Prepared according to Procedure-B, using 1,8-diaminonaphthalene (0.75 mmol, 118 mg, 1.5 equiv) as a diamine. Product (**12b**) was isolated in (133 mg, 91% yield) as a white solid by silica gel chromatography (EtOAc/Hexane = 15:85). A 5-gram reaction gave product **12b** in (3.588 gr, 80% yield).

R_f = 0.45 (15% EtOAc in Hexane).

mp: 185 - 188 °C.

^{1}H NMR (400 MHZ, $CDCl_3$) δ 7.07 (dd, J_1 = 8.3 Hz, J_2 = 7.3 Hz, 2H), 6.98 (dd, J_1 = 8.4 Hz, J_2 = 1.0 Hz, 2H), 6.26 (dd, J_1 = 7.3 Hz, J_2 = 1.1 Hz, 2H), 6.18 (s, 2H), 1.29 (s, 12H).

^{13}C{^{1}H} NMR (101 MHz, $CDCl_3$) : 140.7, 136.5, 127.7, 121.2, 117.7, 105.6, 83.4, 25.2.

^{11}B NMR (128 MHz, $CDCl_3$) δ 28.3, 32.0.

The spectral data are consistent with those reported in the literature.[3]

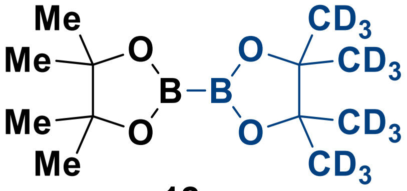

12c

4,4,5,5-tetramethyl-4',4',5',5'-tetrakis(methyl-d3)-2,2'-bi(1,3,2-dioxaborolane):

Prepared according to Procedure-B, using pinacol-d_{12} (0.75 mmol, 98 mg, 1.5 equiv) as a diol. Product (**12c**) was isolated in (118 mg, 89 % yield) as a white solid by silica gel chromatography (EtOAc/Hexane = 15:85).

R_f = 0.34 (10% EtOAc in Hexane).

mp: 137 - 141 °C.

^{1}H NMR (400 MHZ, $CDCl_3$) δ 1.25 (s, 12H).

^{13}C{^{1}H} NMR (101 MHz, $CDCl_3$) δ 83.6, 83.2, 25.1, 24.2 (septet, J = 18.2 HZ, CD_3).

^{11}B NMR (128 MHz, $CDCl_3$) δ 30.5.

^{2}H NMR (77 MHz, $CDCl_3$) δ 1.19.

HRMS (Q-TOF) m/z: $[M+H]^+$ Calcd for $C_{12}H_{13}D_{12}B_2O_4$ 267.2685; Found 267.2686.

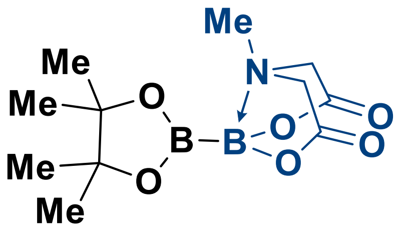

12d

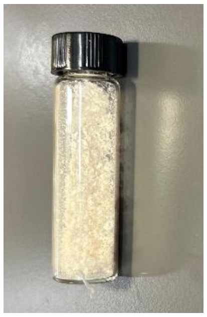

Figure S7. Picture of product **12d** from a large-scale synthesis.

6-methyl-2-(4,4,5,5-tetramethyl-1,3,2-dioxaborolan-2-yl)-1,3,6,2-dioxazaborocane-4,8-dione: Prepared according to Procedure-B, using methyliminodiacetic acid (0.75 mmol, 110 mg, 1.5 equiv). Product (**12d**) was isolated in (95.2 mg, 67% yield) as a white solid by silica gel chromatography (EtOAc/Hexane = 85:15). A 4-gram reaction gave product **12d** in (2.08 gr, 60% yield).

R_f = 0.35 (100% EtOAc).

mp: 240 - 246 °C.

^{1}H NMR (400 MHZ, $CDCl_3$) δ 3.85 (d, *J* = 16.2 Hz, 2H), 3.70 (d, *J* = 16.2 Hz, 2H), 3.02 (s, 3H), 1.23 (s, 12H).

^{13}C{^{1}H} NMR (101 MHz, $CDCl_3$) δ 168.2, 83.6, 61.0, 47.2, 25.1.

^{11}B NMR (128 MHz, $CDCl_3$) δ 33.3, 8.7.

The spectral data are consistent with those reported in the literature.[4]

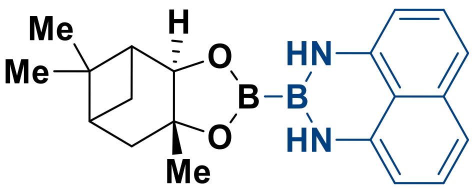

12e

2-((3aR,7aR)-5,5,7a-trimethylhexahydro-4,6-methanobenzo[d][1,3,2]dioxaborol-2-yl)-2,3-dihydro-1H-naphtho[1,8-de][1,3,2]diazaborinine:

Prepared according to Procedure-B, using 1,8-diaminonaphthalene (0.75 mmol, 118 mg, 1.5 equiv) as a diamine. Product (**12e**) was isolated in (133.2 mg, 77 % yield) as a white solid by silica gel chromatography (EtOAc/Hexane = 20:80).

R_f = 0.57 (15% EtOAc in Hexane).

mp: 160 - 163 °C.

^{1}H NMR (400 MHZ, $CDCl_3$) δ 7.07 (dd, J_1 = 8.3 Hz, J_2 = 7.3 Hz, 2H), 6.98 (dd, J_1 = 8.4 Hz, J_2 = 1.0 Hz, 2H), 6.27 (dd, J_1 = 7.3 Hz, J_2 = 1.1 Hz, 2H), 6.19 (s, 2H), 4.29 (dd, J_1 = 8.8 Hz, J_2 =1.9 Hz, 1H), 2.42 – 2.32 (m, 1H), 2.27 – 2.19 (m, 1H), 2.08 (dd, J_1 = 6.0 Hz, J_2 = 5.0 Hz, 1H), 1.97 – 1.85 (m, 2H), 1.43 (s, 3H), 1.31 (s, 3H), 1.10 (d, J = 10.9 Hz, 1H), 0.88 (s, 3H).

^{13}C{^{1}H} NMR (101 MHz, $CDCl_3$) δ 140.6, 136.5, 127.6, 121.2, 117.7, 105.5, 51.2, 39.7, 38.0, 35.3, 28.9, 27.1, 26.6, 24.0.

^{11}B NMR (128 MHz, $CDCl_3$) δ 28.4.

HRMS (Q-TOF) m/z: $[M+H]^+$ Calcd for $C_{20}H_{25}B_2N_2O_2$ 347.2090; Found 347.2096.

2.4 Procedure-C and Characterizations for Products (13a).

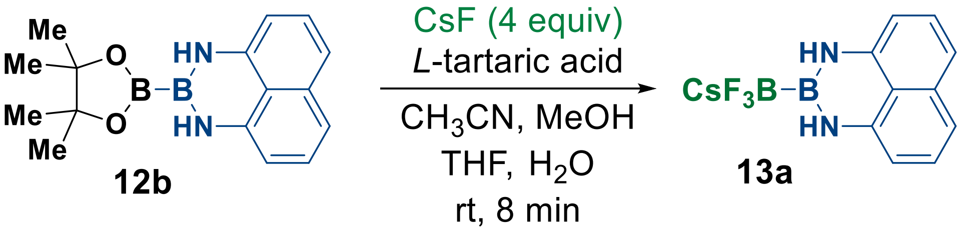

Procedure-C: The reaction took place in an open air environment. Bpin-Bdan (**12b**) (1 mmol, 300 mg, 1 equiv) was dissolved in solution of acetonitrile (3 mL), methanol (3 mL) and THF (0.5 mL). To the above mixture, a solution of CsF (4 mmol, 600 mg, in H_2O 0.2 mL) was added, and the mixture was stirred at room temperature for 1 minutes. Next *L*-tartaric acid (2.04 mmol, 306 mg, in 4 mL THF) was added dropwise to the rapidly stirred solution, during that time a white precipitate crashed out. After 8 minutes, the reaction mixture was filtered to remove the white precipitate and washed thoroughly with excess of acetonitrile (8 mL), then the filtrate was concentrated in a rotary evaporator to give a crude solid. Then washing with diethyl ether and hexane, furnished the corresponding Bdan-BF_3Cs (**13a**) as a white solid via filtration.

Note: Product Bdan-BF_3Cs (**13a**) can be used immediately after isolation for the next step **Figure S8** or kept under Nitrogen atmosphere while protected from light, otherwise compound starts to decompose while changing color to purple **Figure S9**. To remove trace amounts of solvents (Et_2O and Hexane) the salt was left to dry in a Nitrogen (N_2) filled Glovebox, while protected from light.

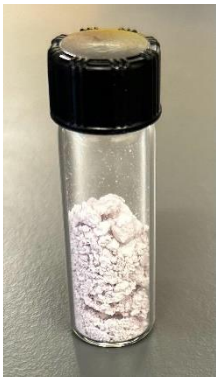

Figure S8. Picture of product **13a** immediately after filtration.

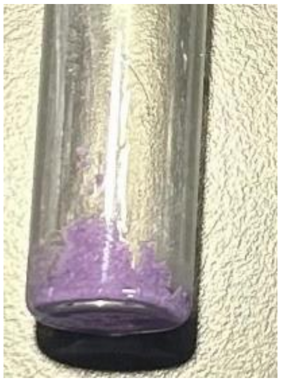

Figure S9. Picture of product **13a** after exposing to light and moisture.

2-(trifluoro-4-boranyl)-2,3-dihydro-1H-naphtho[1,8-de][1,3,2]diazaborinine, cesium salt:
Prepared according to procedure C, product (**13a**) was isolated in (349 mg, 95% yield) as a white solid. A 3-gram reaction gave product **13a** in (3.304 gr, 88% yield).

mp: 190 °C (decomposition).

^{1}H NMR (400 MHZ, DMSO-d_6) δ 7.10 (s, 2H), 6.93 (t, J = 7.4 Hz, 2H), 6.70 (dd, J_1 = 8.3 Hz, J_2 = 1.0 Hz, 2H), 6.36 (dd, J_1 = 7.4 Hz, J_2 = 1.1 Hz, 2H).

^{13}C{^{1}H} NMR (101 MHz, DMSO-d_6) δ 141.2, 134.4, 125.8, 118.2, 112.6, 102.3.

^{11}B NMR (128 MHz, DMSO-d_6) δ 34.9 (brs), 3.6 (s).

^{19}F NMR (376 MHz, DMSO-d_6) δ -128.8 (s).

HRMS (Q-TOF) m/z: $[M+H]^+$ Calcd $C_{10}H_9B_2F_3N_2$ 236.0864; Found 236.1855.

2.5 Procedure-D and Characterizations for Products (14).

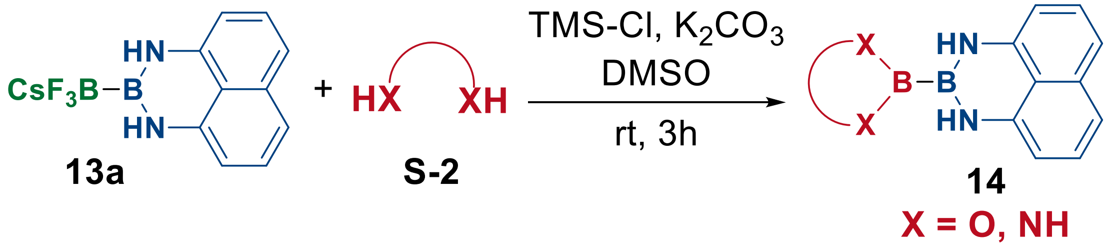

Procedure-D: Inside an oven-dried 25 mL Schleck tube, Bdan-BF_3Cs diborane (**13a**) (0.5 mmol, 1 equiv, 184 mg), K_2CO_3 (1.5 mmol, 3 equiv, 207 mg), and diol/diamine (0.75 mmol, 1.5 equiv) were added and dissolved in dry-DMSO (6 mL) under nitrogen (N_2) protection. To the above mixture, trimethylsilyl chloride TMS-Cl (1.5 mmol, 3 equiv, 190 µL) was added dropwise, and the reaction was left stirring for 3h at room temperature. After completion of the reaction, EtOAc (10 ml) and H_2O (5 ml) were added, and the residue was extracted with EtOAc (3 × 8 mL). The organic layers were combined and washed with brine, dried over $MgSO_4$, and concentrated under reduced pressure using evaporator to obtain a crude material. The crude material was further purified by a short column on silica gel, resulting in the formation of product (**14**).

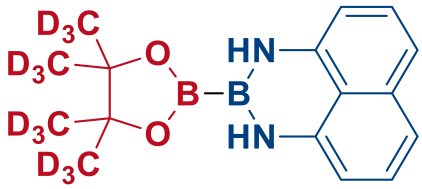

14a

2-(4,4,5,5-tetrakis(methyl-d3)-1,3,2-dioxaborolan-2-yl)-2,3-dihydro-1H-naphtho[1,8-de][1,3,2]diazaborinine:

Prepared according to general Procedure-D, using pinacol-d_{12} (0.75 mmol, 98 mg, 1.5 equiv) as a diol. Product (**14a**) was isolated in (132 mg, 86% yield) as a white solid by silica gel chromatography (EtOAc/Hexane = 20:80).

R_f = 0.42 (15% EtOAc in Hexane).

mp: 185 - 187 °C.

^{1}H NMR (400 MHZ, $CDCl_3$) δ 7.10 – 7.03 (m, 2H), 6.98 (dd, J_1 = 8.3 Hz, J_2 = 1.0 Hz, 2H), 6.26 (dd, J_1 = 7.3 Hz, J_2 = 1.0 Hz, 2H), 6.19 (s, 2H).

$^{13}C\{^1H\}$ NMR (101 MHz, $CDCl_3$)) δ 140.6, 136.5, 127.6, 121.2, 117.7, 105.6, 83.1, 24.3 (septet, J = 24.2 Hz, CD_3).

^{11}B NMR (128 MHz, $CDCl_3$) δ 31.9, 27.8.

^2H NMR (77 MHz, $CDCl_3$) δ 1.29.

HRMS (Q-TOF) m/z: $[M+H]^+$ Calcd for $C_{16}H_9D_{12}B_2N_2O_2$ 307.2540; Found 307.2536.

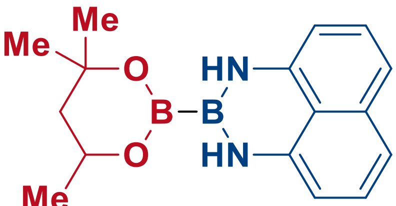

14b

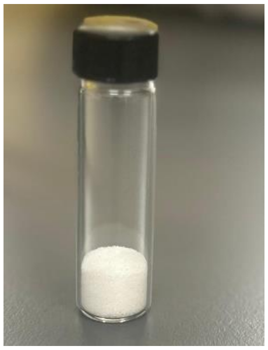

Figure S10. Pictures of product **14b** from a large-scale synthesis.

2-(4,4,6-trimethyl-1,3,2-dioxaborinan-2-yl)-2,3-dihydro-1H-naphtho[1,8-de][1,3,2]diazaborinine:

Prepared according to general Procedure-D, using hexylene-glycole (0.75 mmol, 88 mg, 1.5 equiv) as a diol. Product (**14b**) was isolated in (122 mg, 83% yield) as a white solid by silica gel chromatography (EtOAc/Hexane = 20:80). A 2-gram reaction gave product **14b** in (1.203 gr, 75% yield).

R_f = 0.50 (15% EtOAc in Hexane).

mp: 155 - 156 °C.

^1H NMR (400 MHZ, $CDCl_3$) δ 7.06 (dd, J_1 = 8.3 Hz, J_2 = 7.4 Hz, 2H), 6.96 (dd, J_1 = 8.4 Hz, J_2 = 1.0 Hz, 2H), 6.27 (dd, J_1 = 7.4 Hz, J_2 = 1.1 Hz, 2H), 6.13 (s, 2H), 4.22 – 4.14 (m, 1H), 1.82 (dd, J_1 = 13.8, Hz, J_2 = 3.0 Hz, 1H), 1.57 – 1.48 (m, 1H), 1.32 (s, 6H), 1.29 (d, J = 6.2 Hz, 3H).

$^{13}C\{^1H\}$ NMR (101 MHz, $CDCl_3$) δ 141.1, 136.6, 127.6, 121.1, 117.3, 105.3, 70.3, 64.2, 46.4, 31.4, 28.6, 23.3.

^{11}B NMR (128 MHz, $CDCl_3$) δ 29.0.

HRMS (Q-TOF) m/z: $[M+H]^+$ $C_{16}H_{21}B_2O_2N_2$ 295.1784; Found 295.1783.

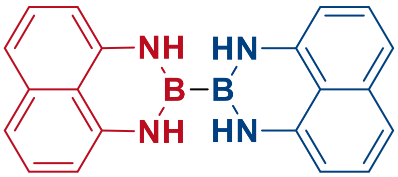

14c

1H,1'H,3H,3'H-2,2'-binaphtho[1,8-de][1,3,2]diazaborinine:

Prepared according to general Procedure-D, using 1,8-diaminonaphthalene (0.75 mmol, 118 mg, 1.5 equiv) as a diamine. Product (**14c**) was isolated in (146 mg, 88% yield) as a white solid by silica gel chromatography (EtOAc/Hexane = 25:75).

R_f = 0.42 (15% EtOAc in Hexane).

mp: 201 - 205 °C.

^{1}H NMR (400 MHZ, DMSO-d_6) δ 8.10 (s, 2H), 7.08 (dd, J_1 = 8.2 Hz, J_2 = 7.4 Hz, 2H), 6.88 (dd, J_1 = 8.3 Hz, J_2 = 1.0 Hz, 2H), 6.34 (dd, J_2 = 7.4 Hz, J_2 = 1.0 Hz, 2H).

^{13}C{^{1}H} NMR (101 MHz, DMSO-d_6) δ 142.1, 136.6, 128.3, 120.9, 116.6, 105.1.

^{11}B NMR (128 MHz, DMSO-d_6) δ 28.4.

The spectral data are consistent with those reported in the literature.[5]

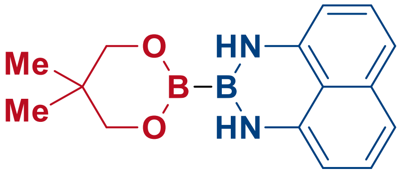

14d

2-(5,5-dimethyl-1,3,2-dioxaborinan-2-yl)-2,3-dihydro-1H-naphtho[1,8-de][1,3,2]diazaborinine:

Prepared according to general Procedure-D, using 2,2-dimethyl-1,3-propanediol (0.75 mmol, 78 mg, 1.5 equiv) as a diol. Product (**14d**) was isolated in (88 mg, 63% yield) as a white solid by silica gel chromatography (EtOAc/Hexane = 20:80).

R_f = 0.37 (15% EtOAc in Hexane).

mp: 170 - 173 °C.

^{1}H NMR (400 MHZ, $CDCl_3$) δ 7.07 (dd, J_1 = 8.3 Hz, J_2 = 7.3 Hz, 2H), 6.97 (dd, J_1 = 8.4 Hz, J_2 = 1.0 Hz, 2H), 6.26 (dd, J_1 = 7.3 Hz, J_2 = 1.0 Hz, 2H), 6.15 (s, 2H), 3.61 (s, 4H), 0.99 (s, 6H).

^{13}C{^{1}H} NMR (126 MHz, $CDCl_3$) δ 141.0, 136.6, 127.7, 121.2, 117.4, 105.4, 71.5, 31.8, 22.2.

^{11}B NMR (128 MHz, $CDCl_3$) δ 28.6.

The spectral data are consistent with those reported in the literature.[6]

3. X-Ray Crystallography Data.

A suitable crystal was selected and subject on a XtaLAB Synergy, Single source at offset/far, HyPix diffractometer. The crystal was kept at 175.0(1) K during data collection. Using Olex2,[7] the structure was solved with the SHELXT[8] structure solution program using Intrinsic Phasing and refined with the SHELXL refinement package using Least Squares Minimization.[9]

Table S2. Crystal data and structure refinement for **(11a)**.

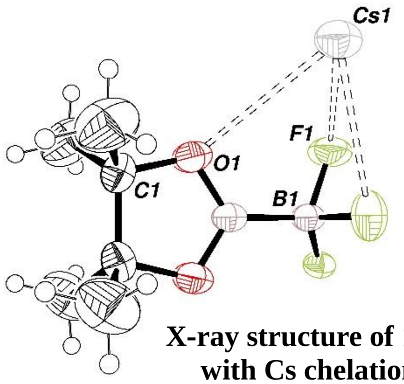

X-ray structure of 11a with Cs chelation CCDC : 2329696

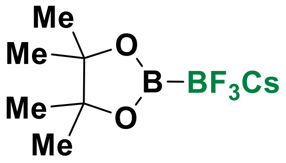

11a

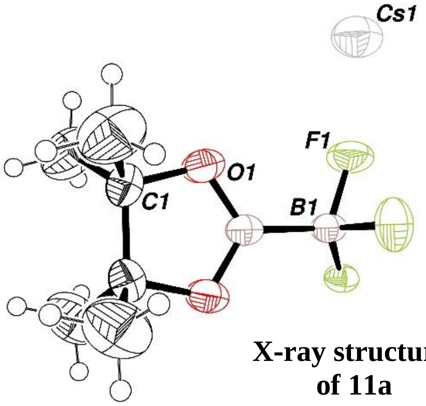

X-ray structure of 11a CCDC : 2329696

The ellipsoid contour is with 50% probability level.

AhmadM43b

Table 1 Crystal data and structure refinement for AhmadM43b.

Identification code	AhmadM43b
Empirical formula	$C_6H_{12}B_2CsF_3O_2$
Formula weight	327.69
Temperature/K	175.0(1)
Crystal system	orthorhombic
Space group	Cmme
a/Å	11.4322(4)
b/Å	9.8359(4)
c/Å	10.0679(5)
α/°	90
β/°	90
γ/°	90
Volume/Å^3	1132.09(8)
Z	4
ρ_{calc}g/cm^3	1.923
μ/mm^{-1}	3.282
F(000)	624.0
Crystal size/mm^3	0.2 × 0.15 × 0.03
Radiation	Mo Kα (λ = 0.71073)
2Θ range for data collection/°	4.046 to 64.3
Index ranges	$-16 \leq h \leq 16$, $-13 \leq k \leq 14$, $-13 \leq l \leq 14$
Reflections collected	8515
Independent reflections	1011 [R_{int} = 0.0286, R_{sigma} = 0.0159]
Data/restraints/parameters	1011/0/75
Goodness-of-fit on F^2	1.170
Final R indexes [I>=2σ (I)]	R_1 = 0.0231, wR_2 = 0.0446
Final R indexes [all data]	R_1 = 0.0389, wR_2 = 0.0501
Largest diff. peak/hole / e Å^{-3}	0.57/-0.36

4. NMR Spectra.

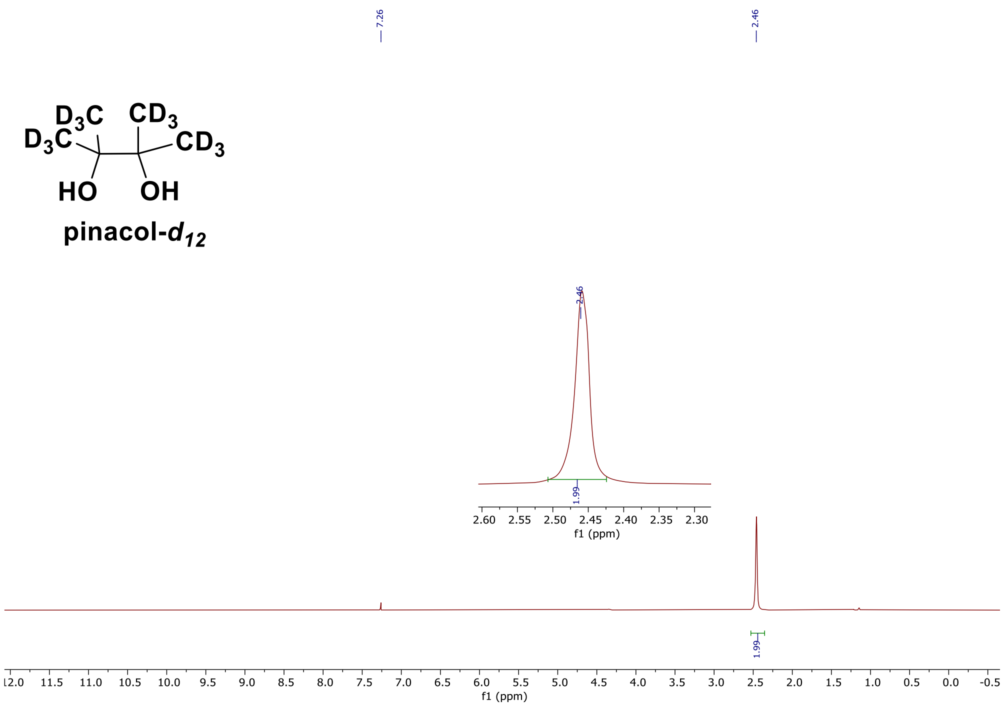

^{1}H NMR (400 MHz, $CDCl_3$) of compound (**pinacol-*d*$_{12}$**).

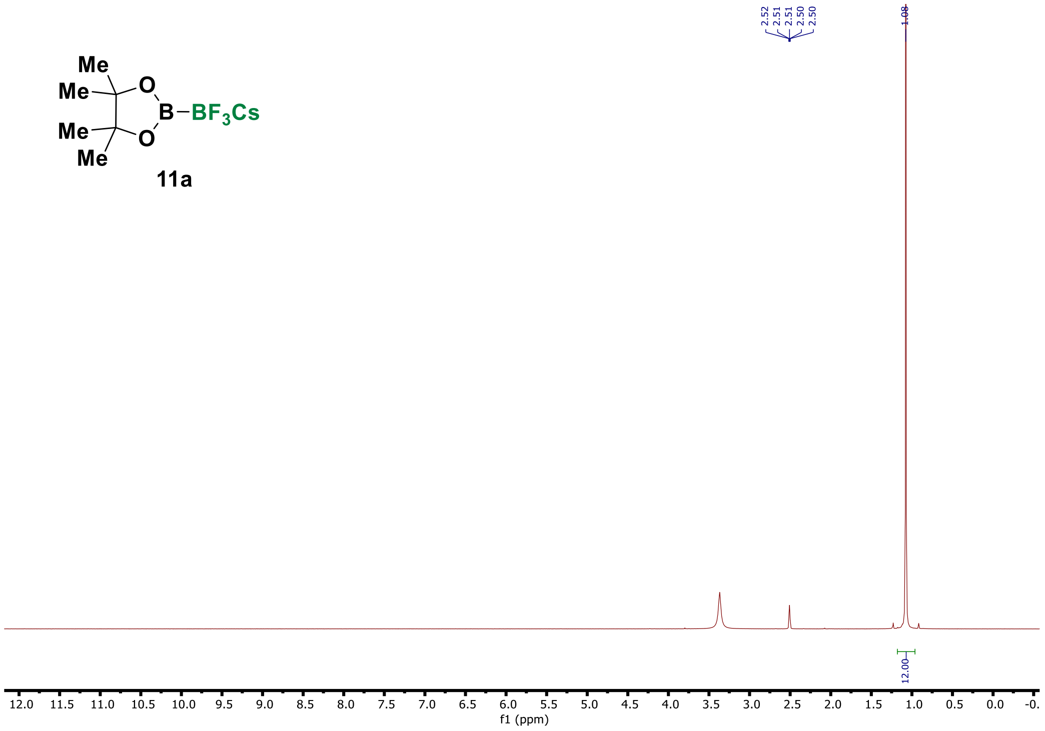

^{1}H NMR (400 MHz, DMSO-d_6) of compound (**11a**).

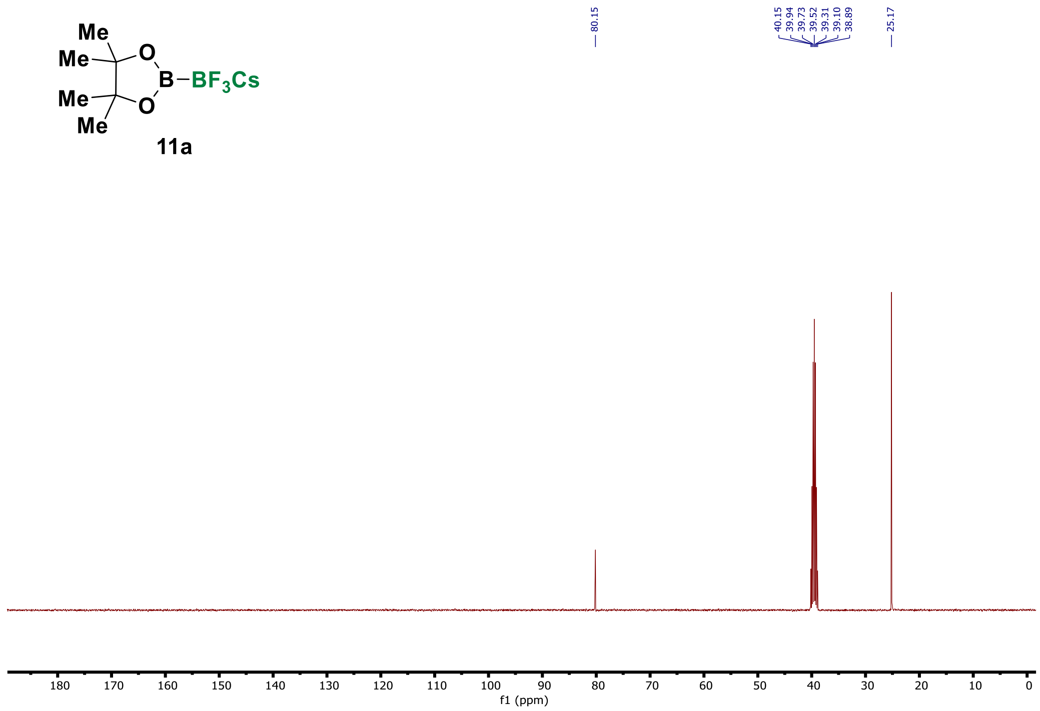

$^{13}C\{^1H\}$ NMR (101 MHz, DMSO-d_6) of compound (**11a**).

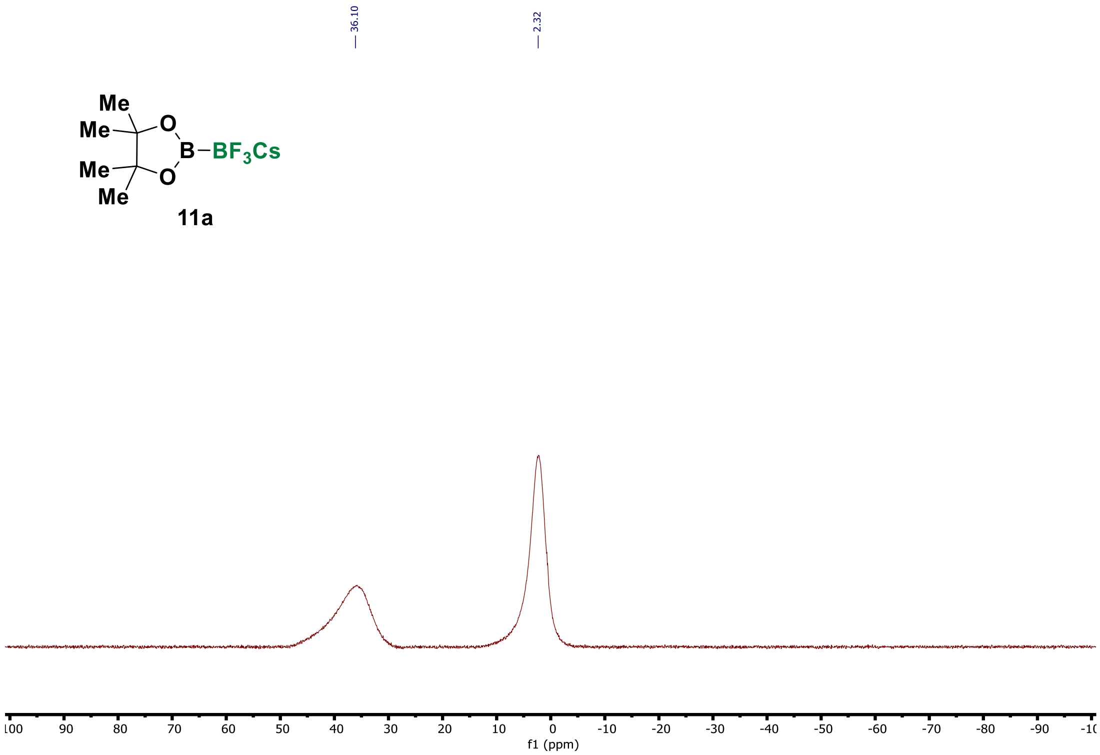

^{11}B NMR (128 MHz, DMSO-d_6) of compound (**11a**).

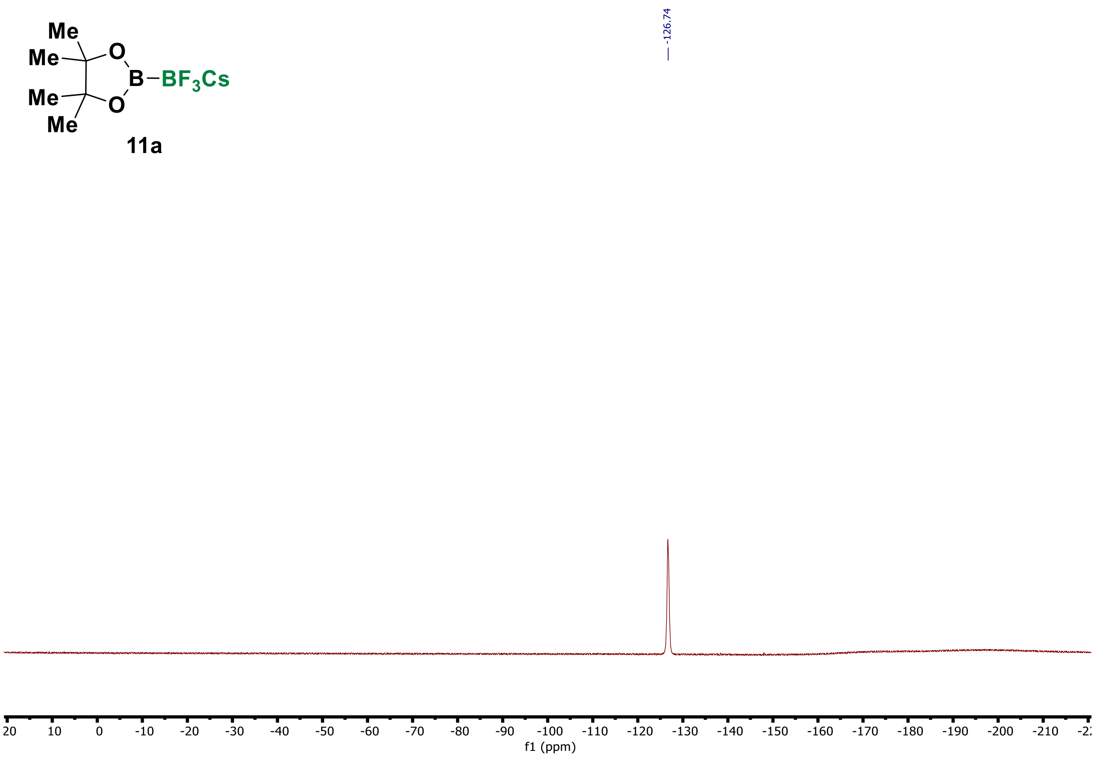

^{19}F NMR (376 MHz, DMSO-d_6) of compound (**11a**).

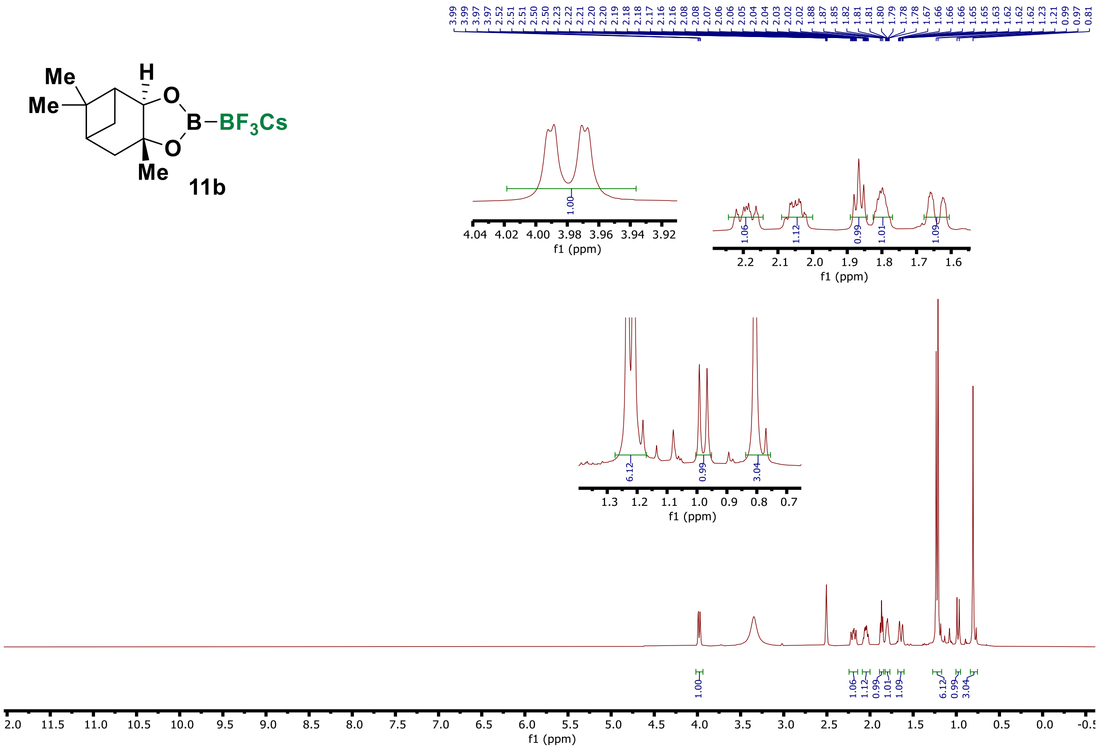

^{1}H NMR (400 MHz, DMSO-d_6) of compound (**11b**).

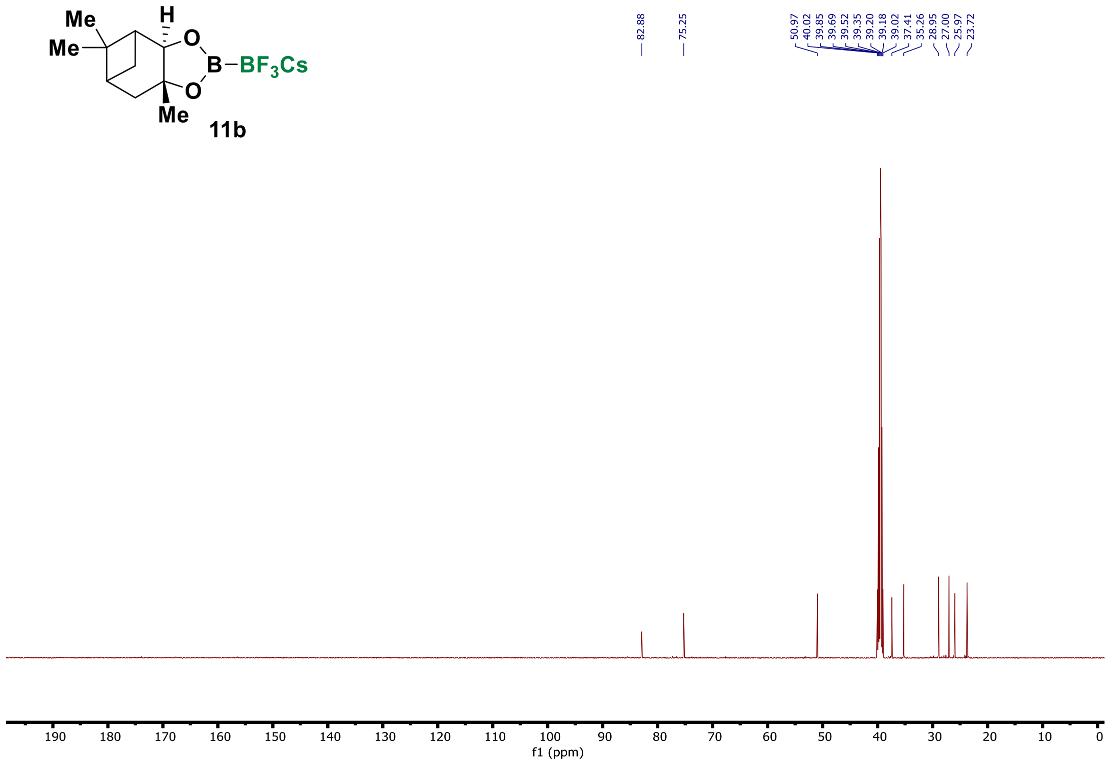

$^{13}C\{^{1}H\}$ NMR (101 MHz, DMSO-d_6) of compound (**11b**).

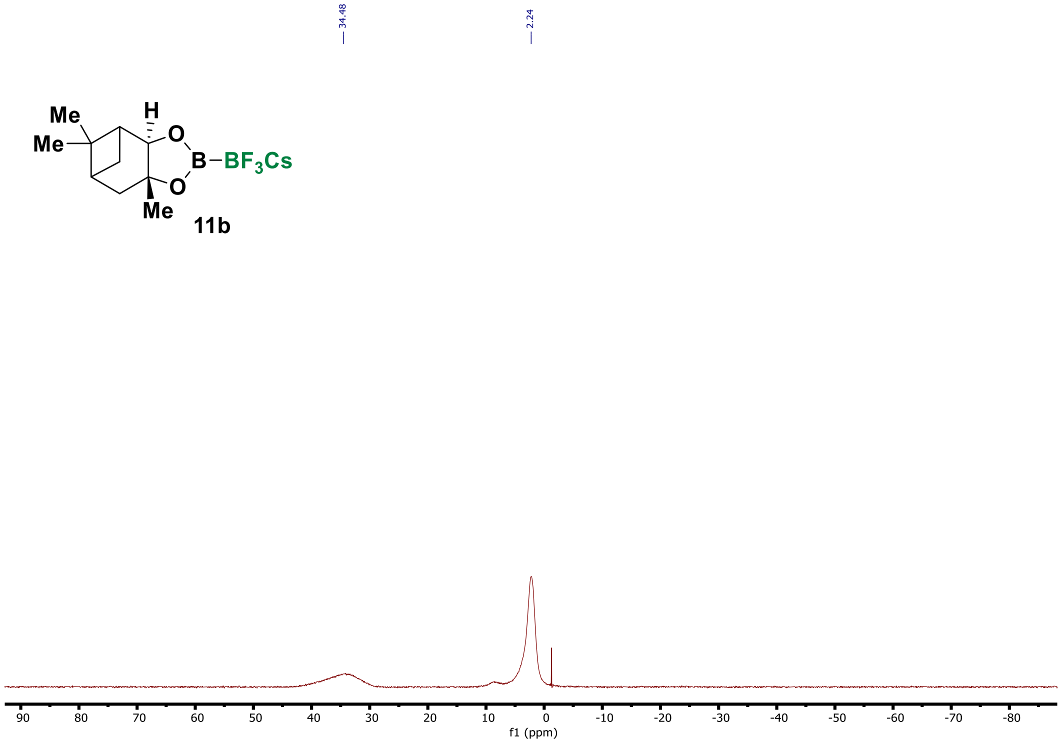

^{11}B NMR (128 MHz, DMSO-d_6) of compound (**11b**).

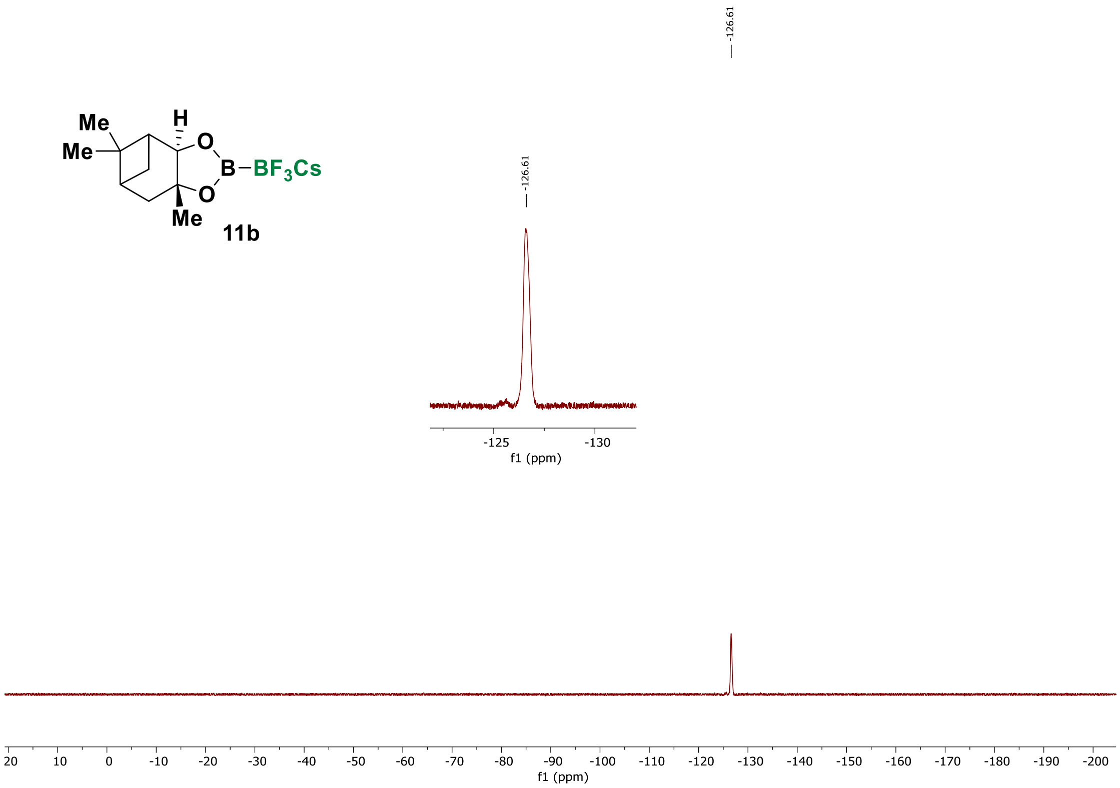

^{19}F NMR (376 MHz, DMSO-d_6) of compound (**11b**).

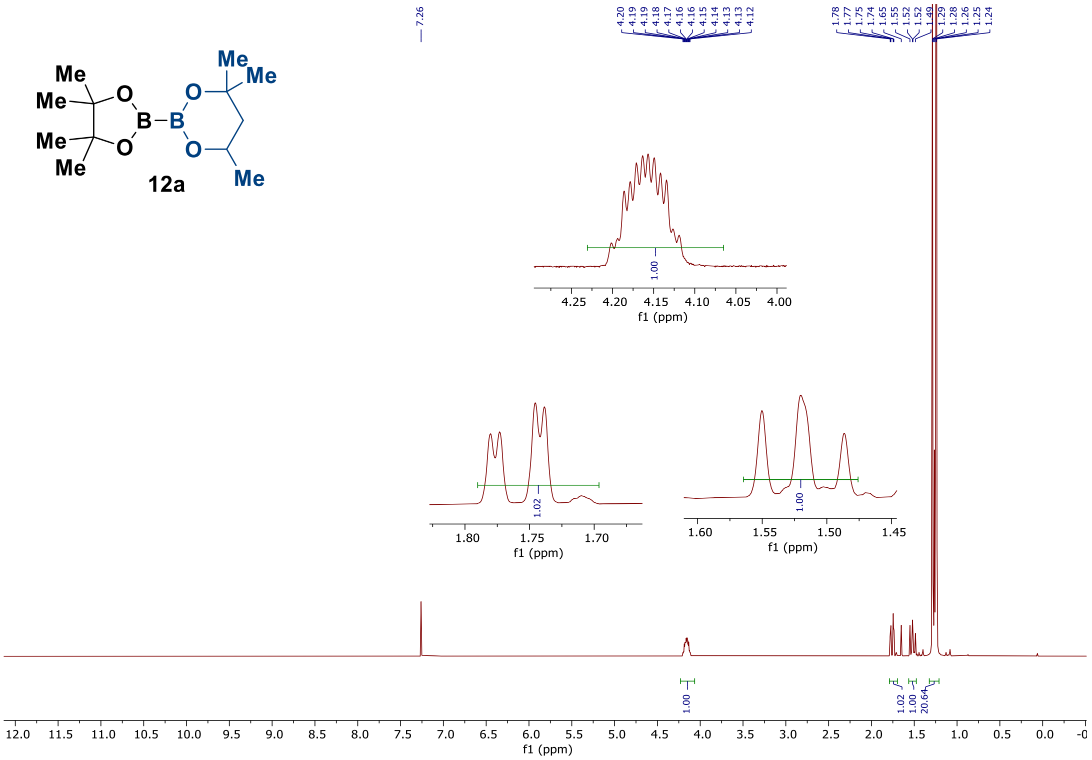

^{1}H NMR (400 MHz, $CDCl_3$) of compound (**12a**).

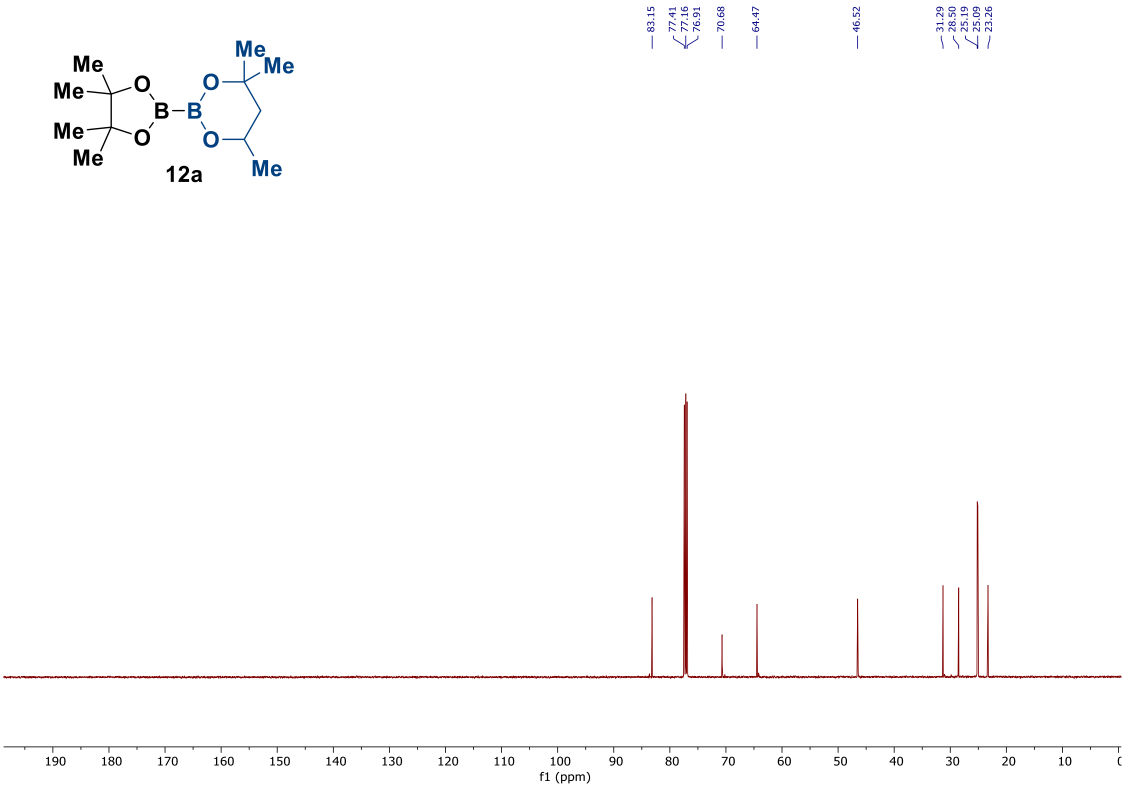

$^{13}C\{^{1}H\}$ NMR (101 MHz, $CDCl_3$) of compound (**12a**).

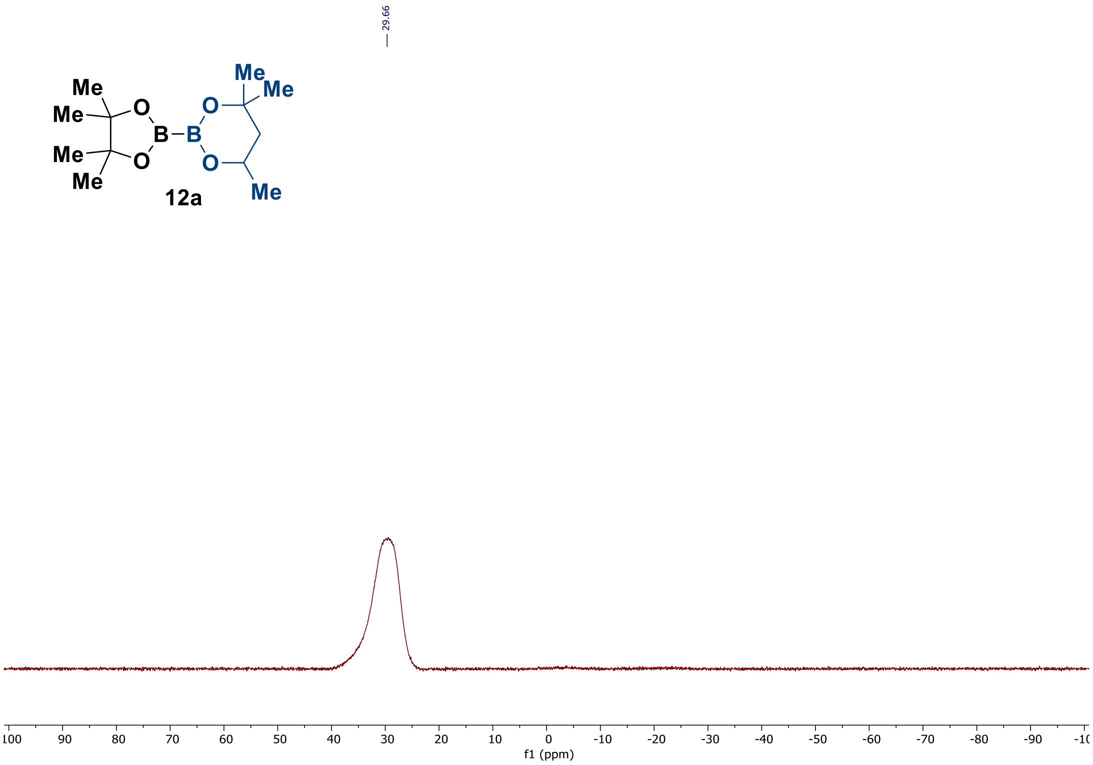

^{11}B NMR (128 MHz, $CDCl_3$) of compound (**12a**).

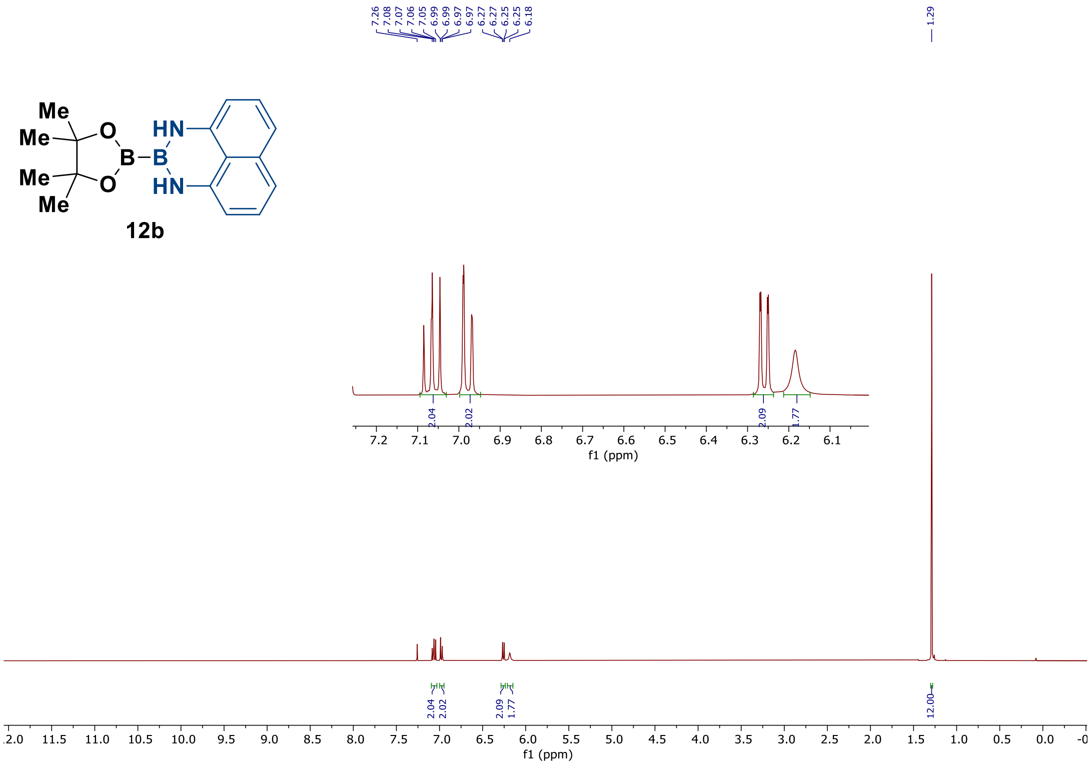

^{1}H NMR (400 MHz, $CDCl_3$) of compound (**12b**).

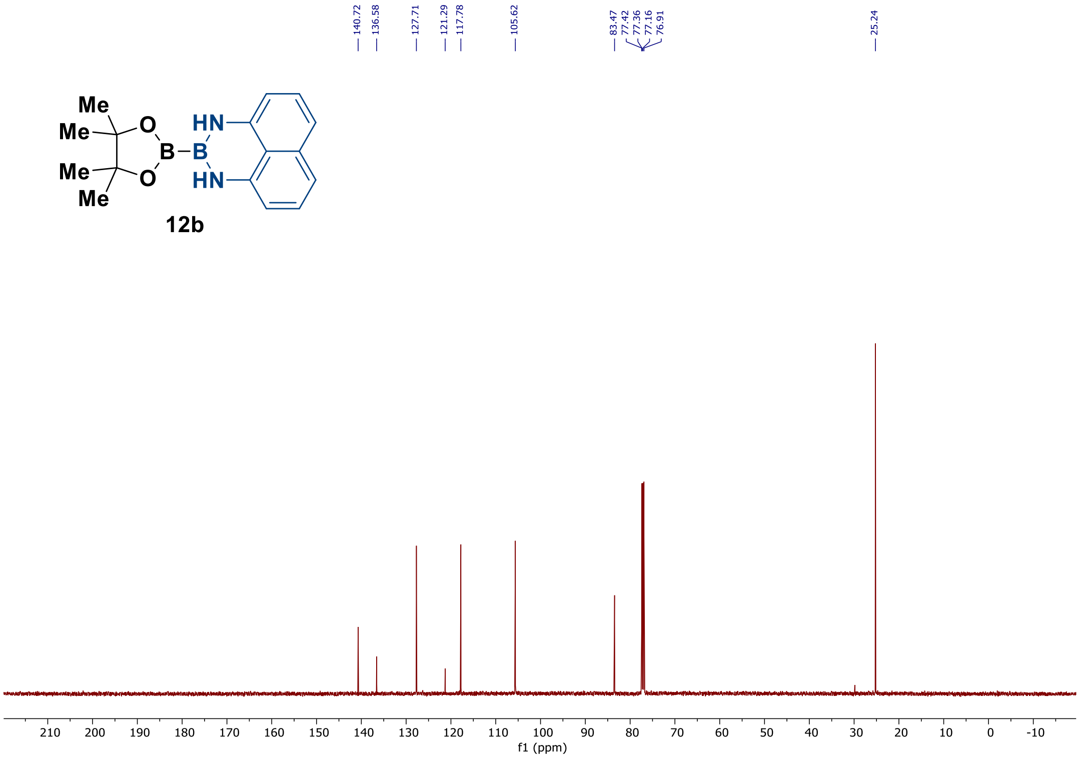

$^{13}C\{^{1}H\}$ NMR (101 MHz, $CDCl_3$) of compound (**12b**).

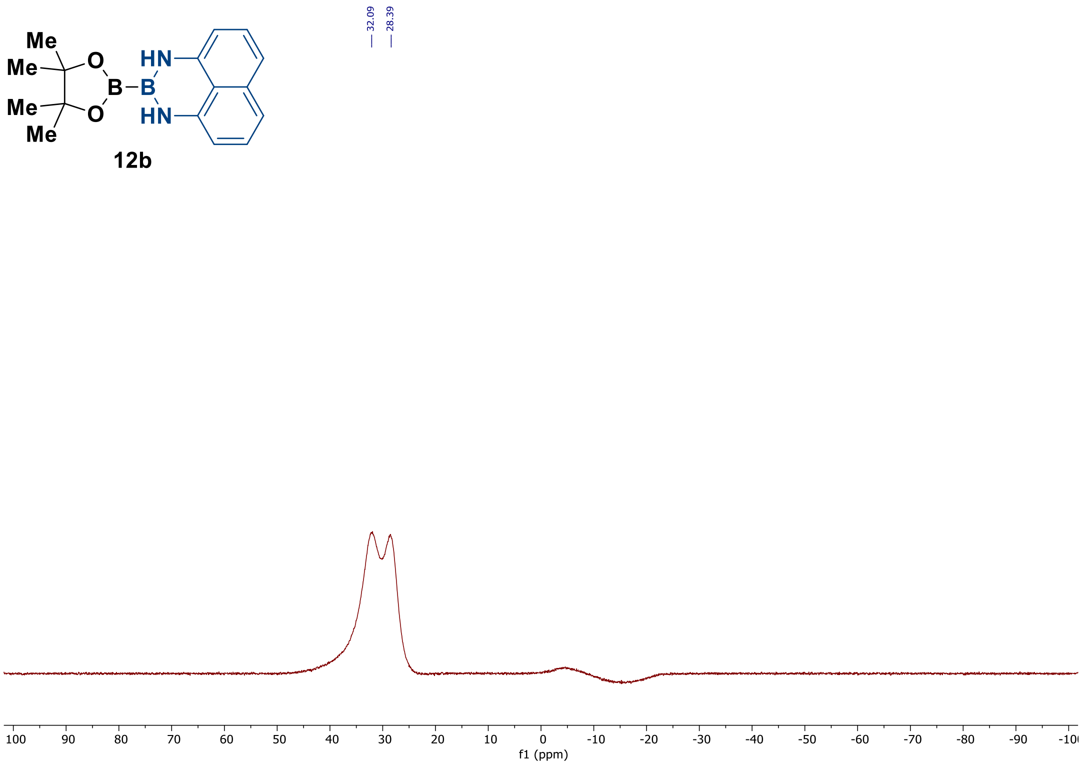

^{11}B NMR (128 MHz, $CDCl_3$) of compound (**12b**).

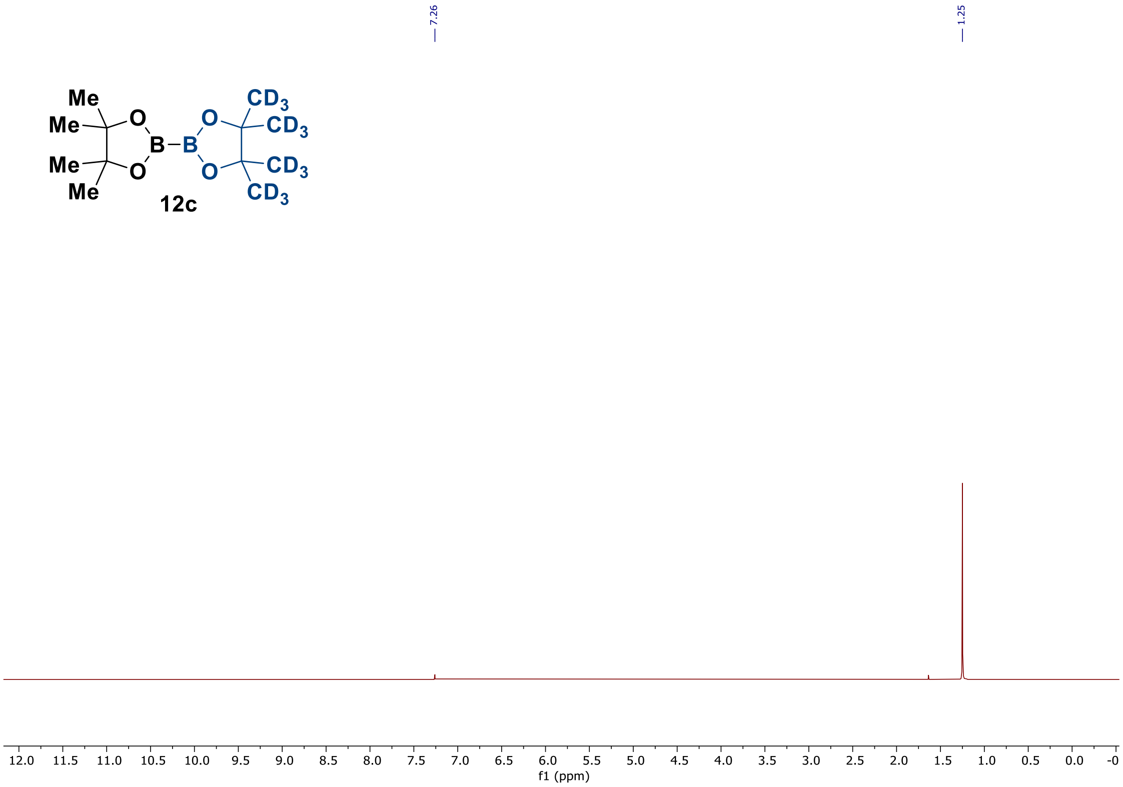

^{1}H NMR (400 MHz, $CDCl_3$) of compound (**12c**).

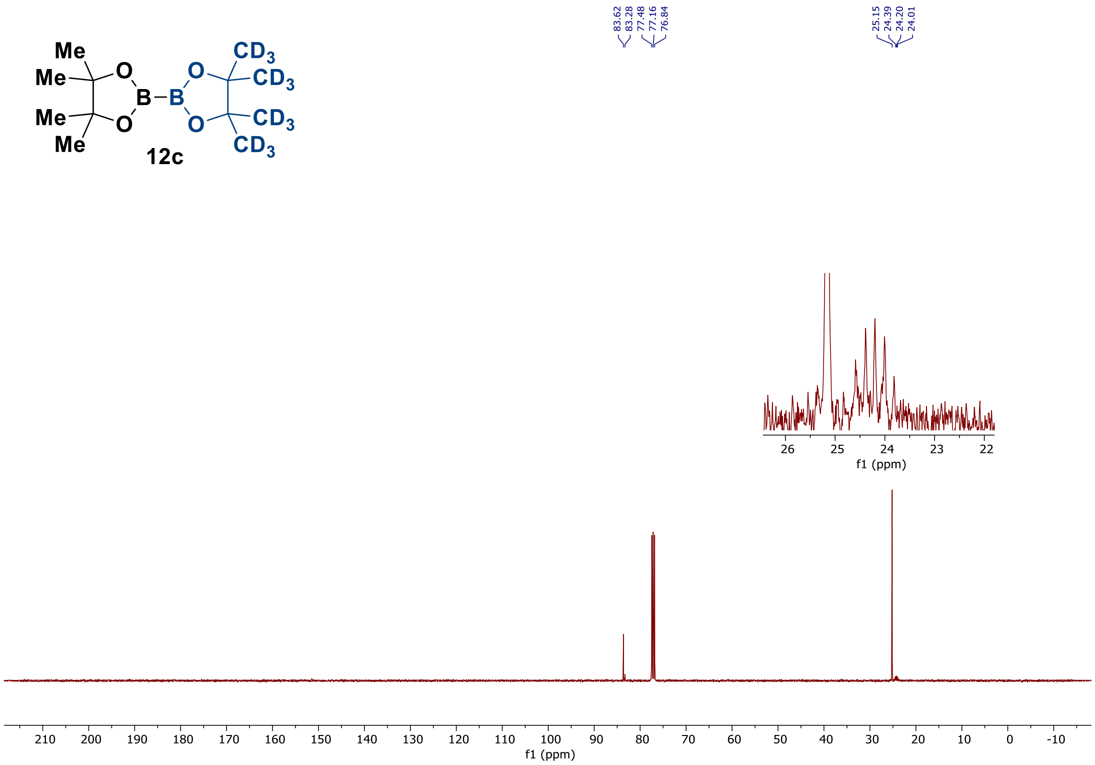

$^{13}C\{^{1}H\}$ NMR (101 MHz, $CDCl_3$) of compound (**12c**).

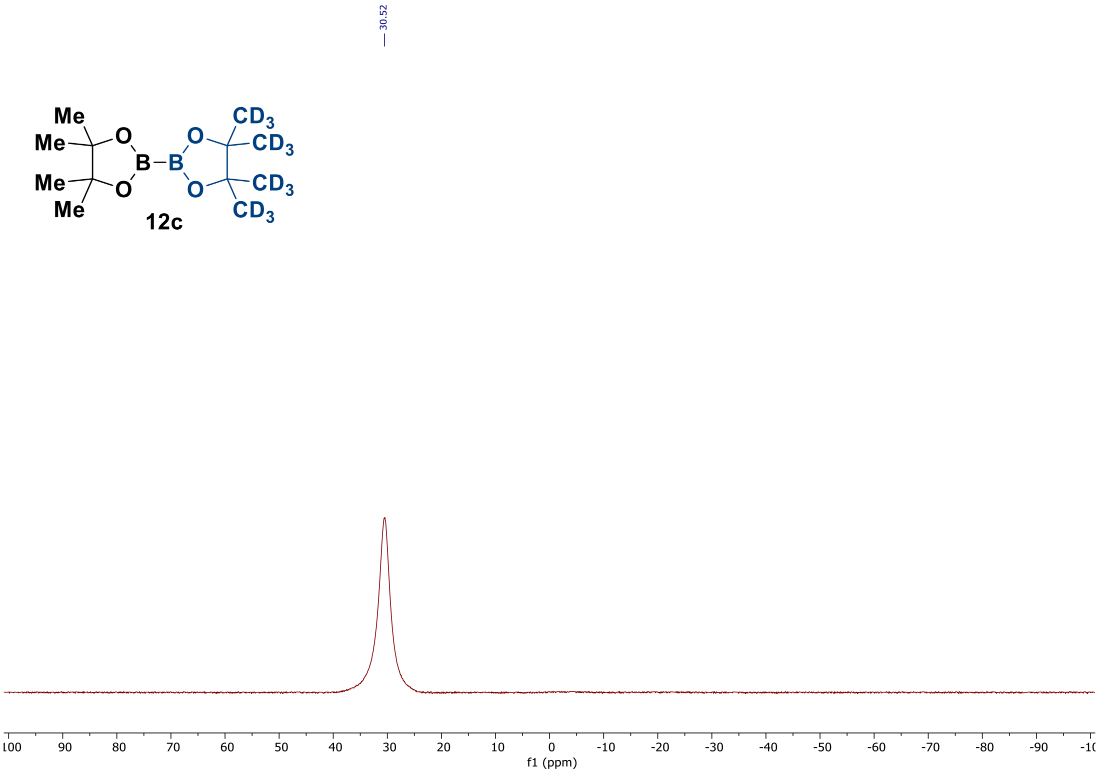

^{11}B NMR (128 MHz, $CDCl_3$) of compound (**12c**).

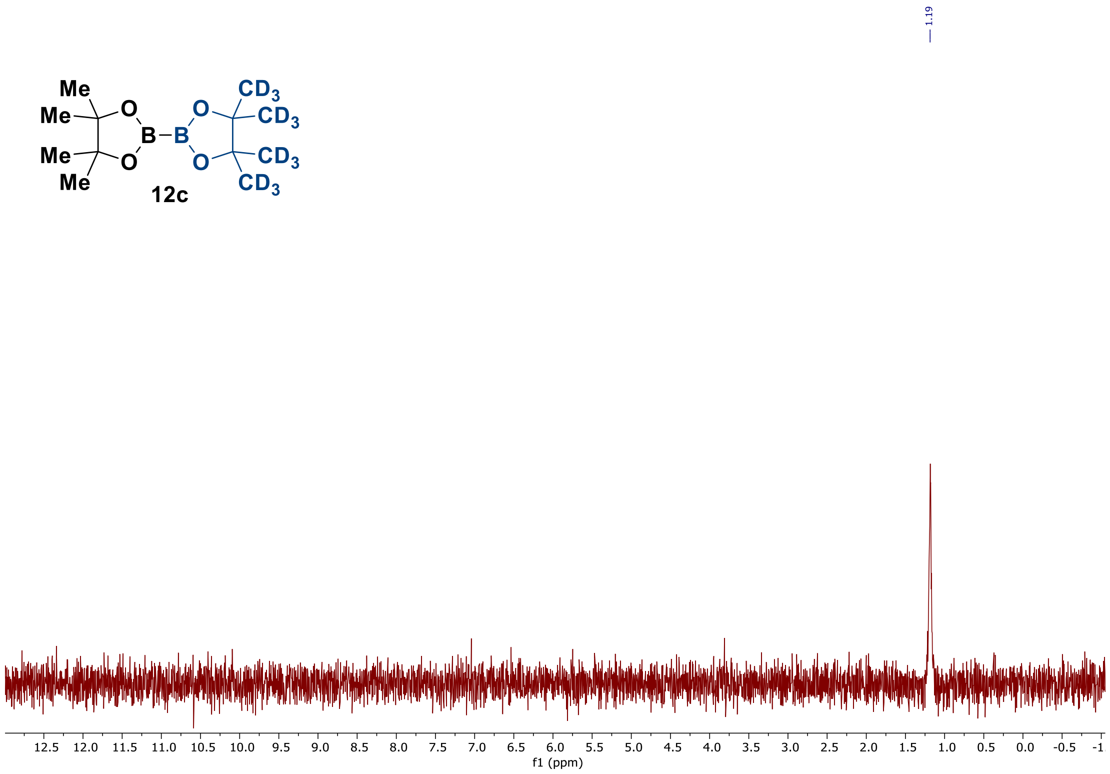

^{2}H NMR (77 MHz, $CHCl_3$) of compound (**12c**).

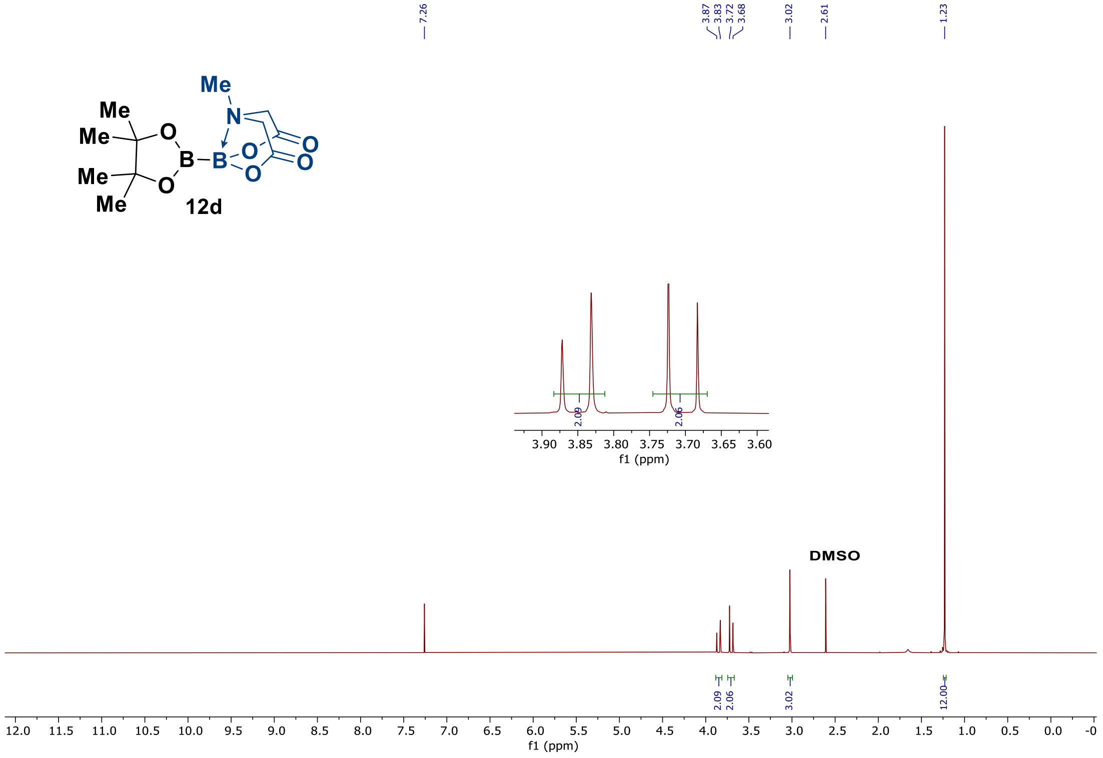

^{1}H NMR (400 MHz, $CDCl_3$) of compound (**12d**).

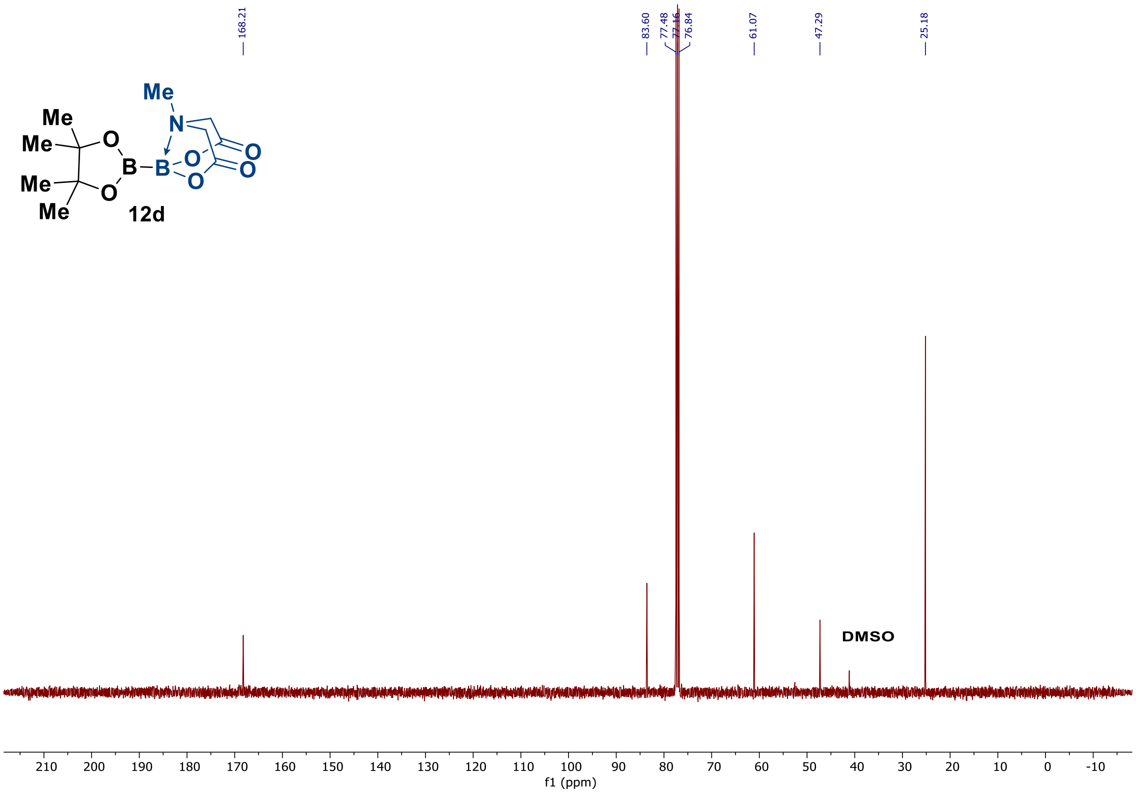

$^{13}C\{^{1}H\}$ NMR (101 MHz, $CDCl_3$) of compound (**12d**).

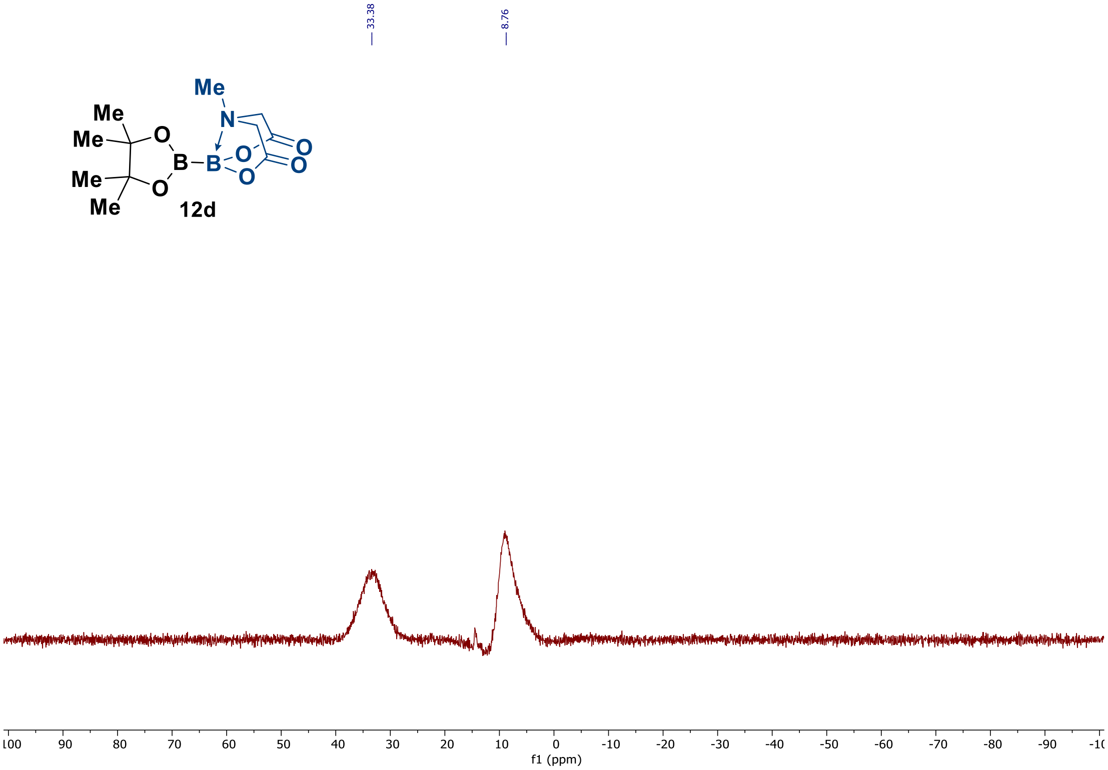

^{11}B NMR (128 MHz, $CDCl_3$) of compound (**12d**).

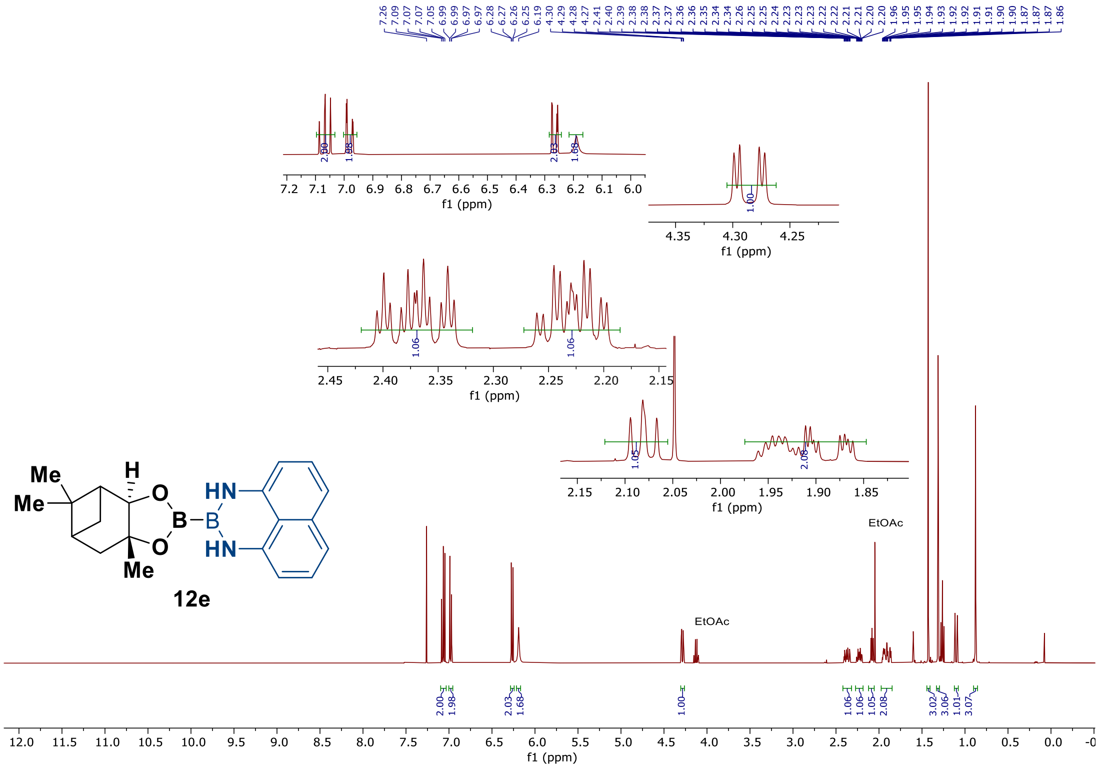

^{1}H NMR (400 MHz, $CDCl_3$) of compound (**12e**).

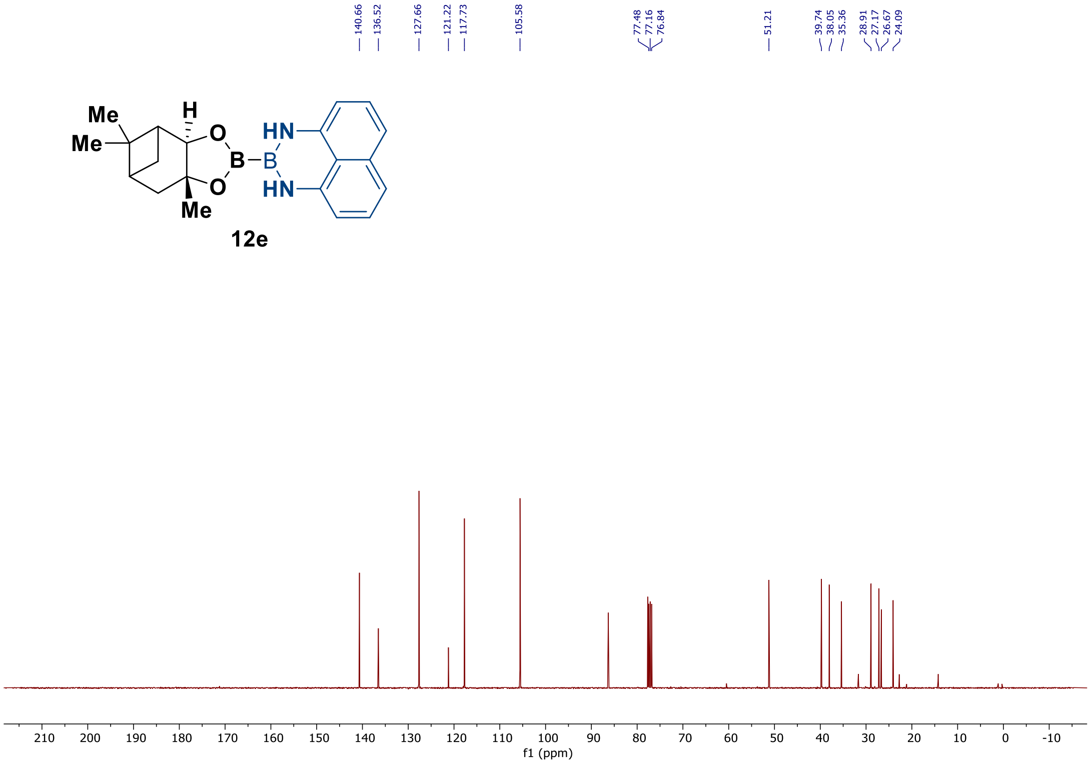

^{13}C{^{1}H} NMR (101 MHz, $CDCl_3$) of compound (**12e**).

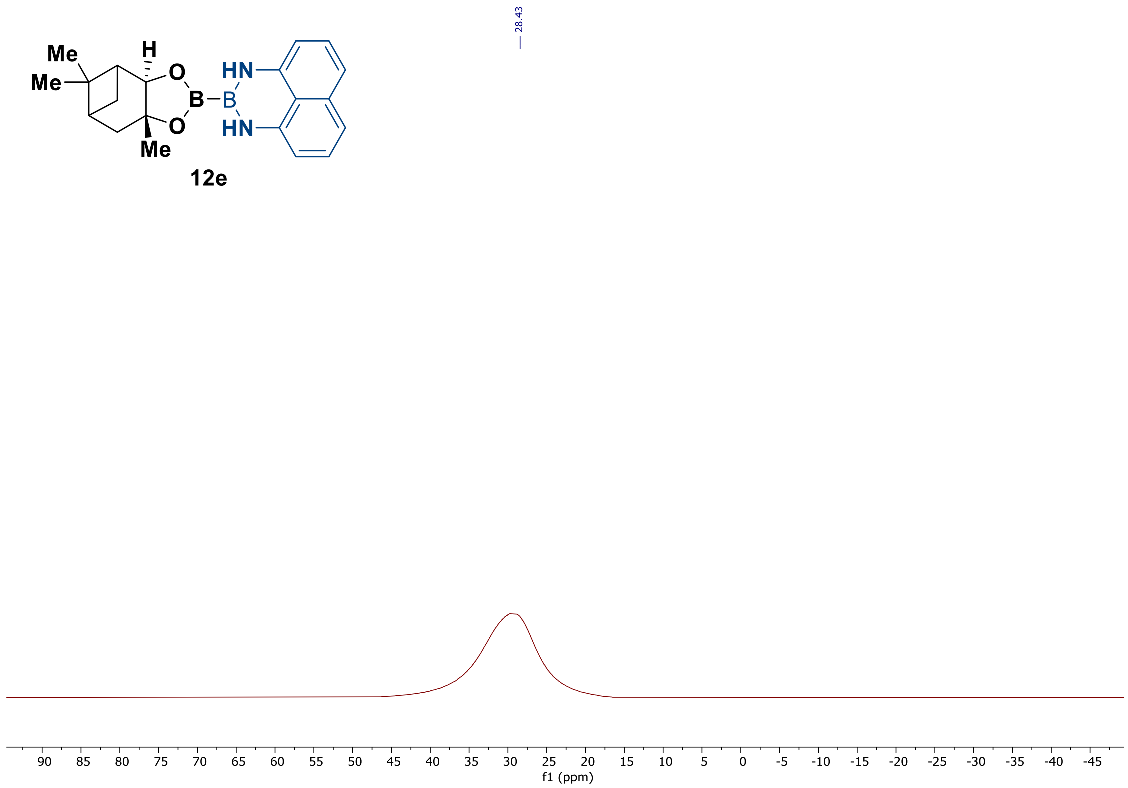

^{11}B NMR (128 MHz, $CDCl_3$) of compound (**12e**).

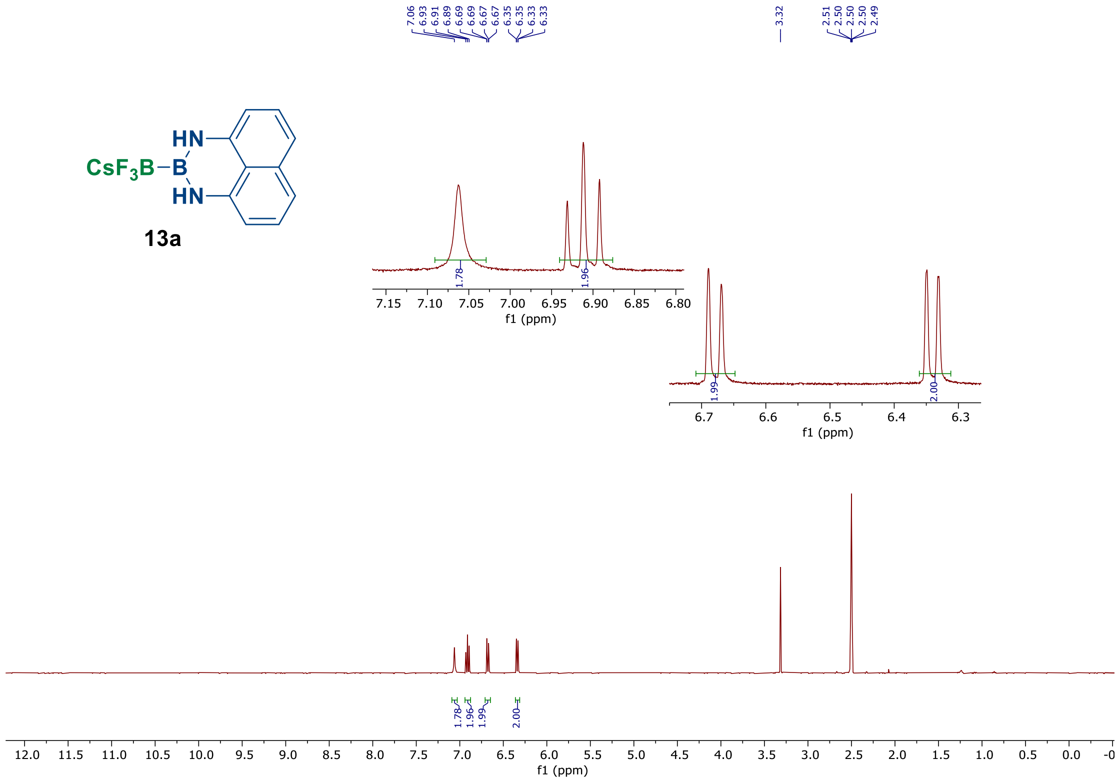

^{1}H NMR (400 MHz, DMSO-d_6) of compound (**13a**).

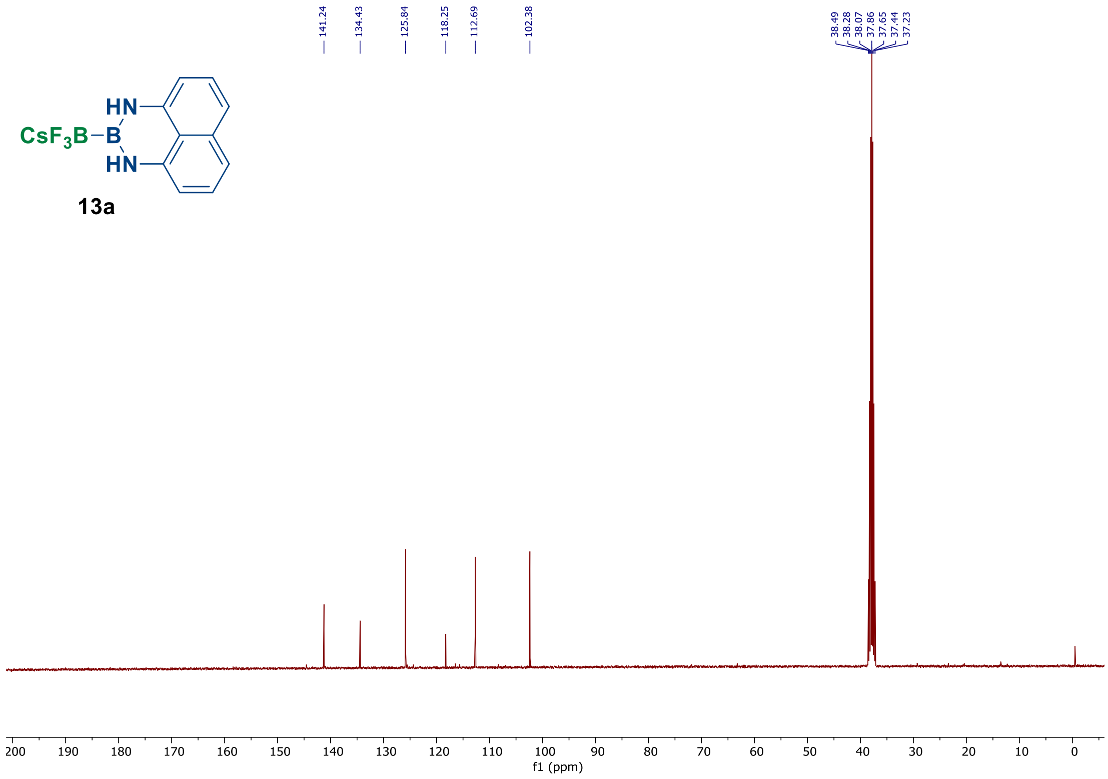

$^{13}C\{^{1}H\}$ NMR (101 MHz, DMSO-d_6) of compound (**13a**).

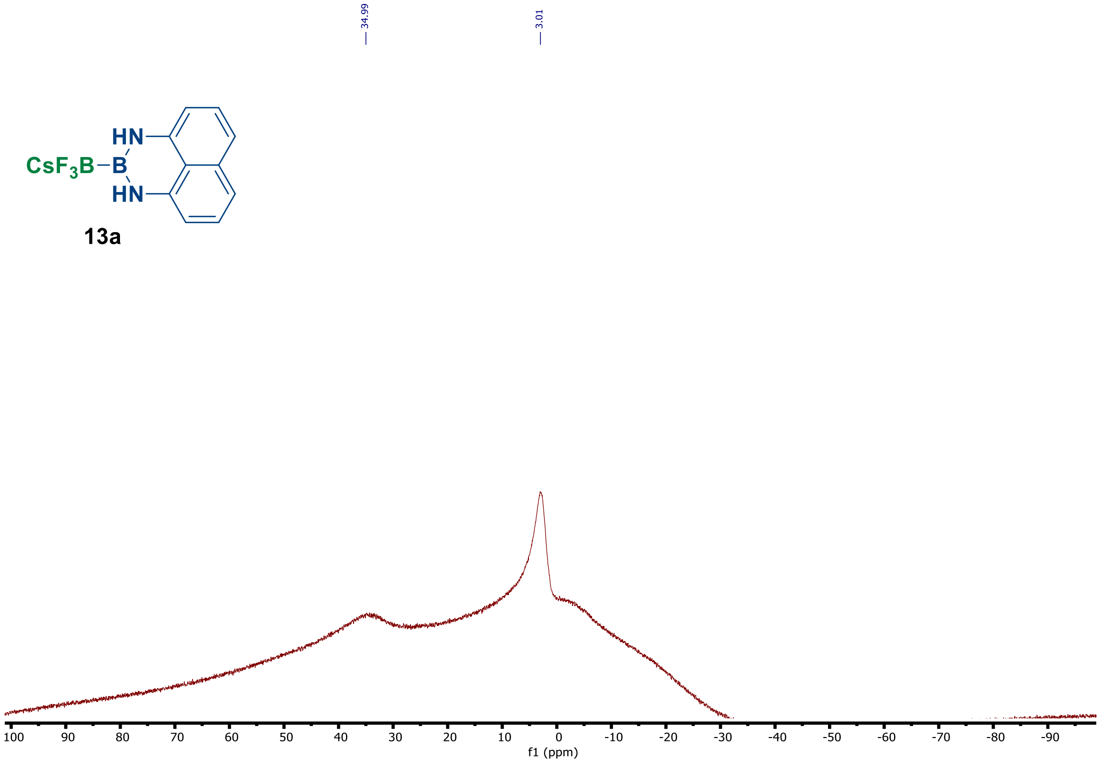

^{11}B NMR (128 MHz, DMSO-d_6) of compound (**13a**).

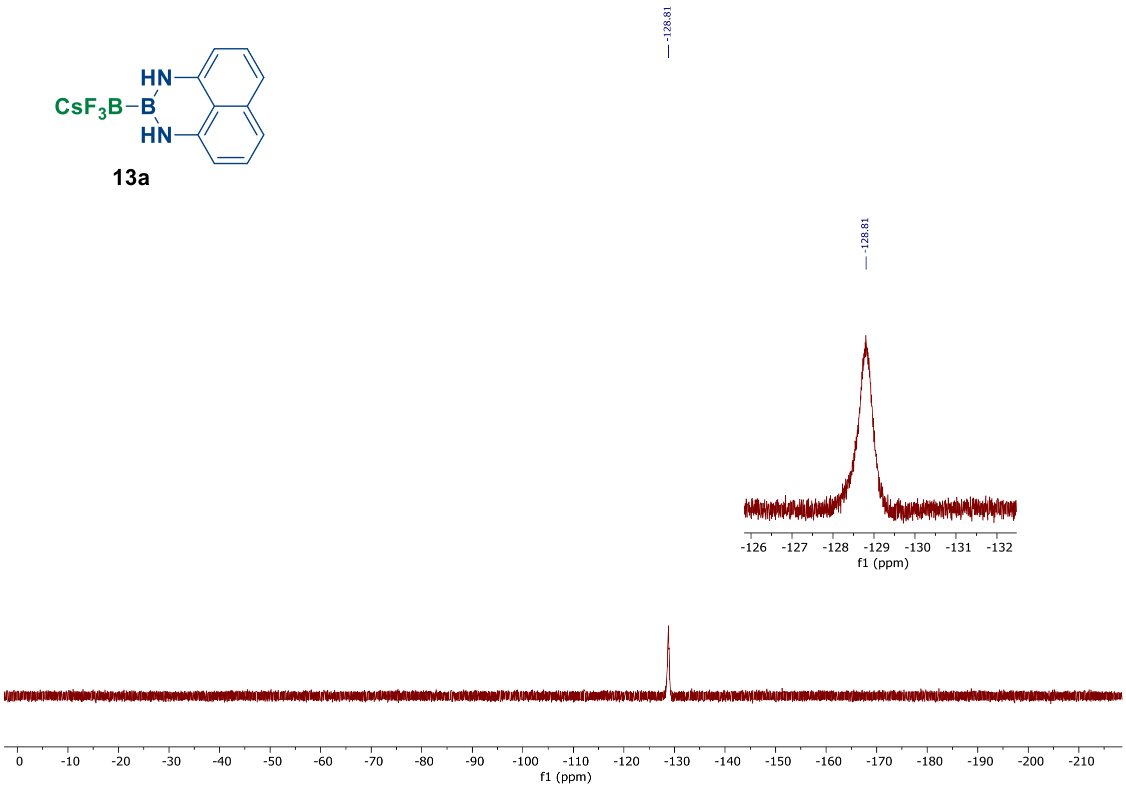

^{19}F-NMR (128 MHz, DMSO-d_6) of compound (**13a**).

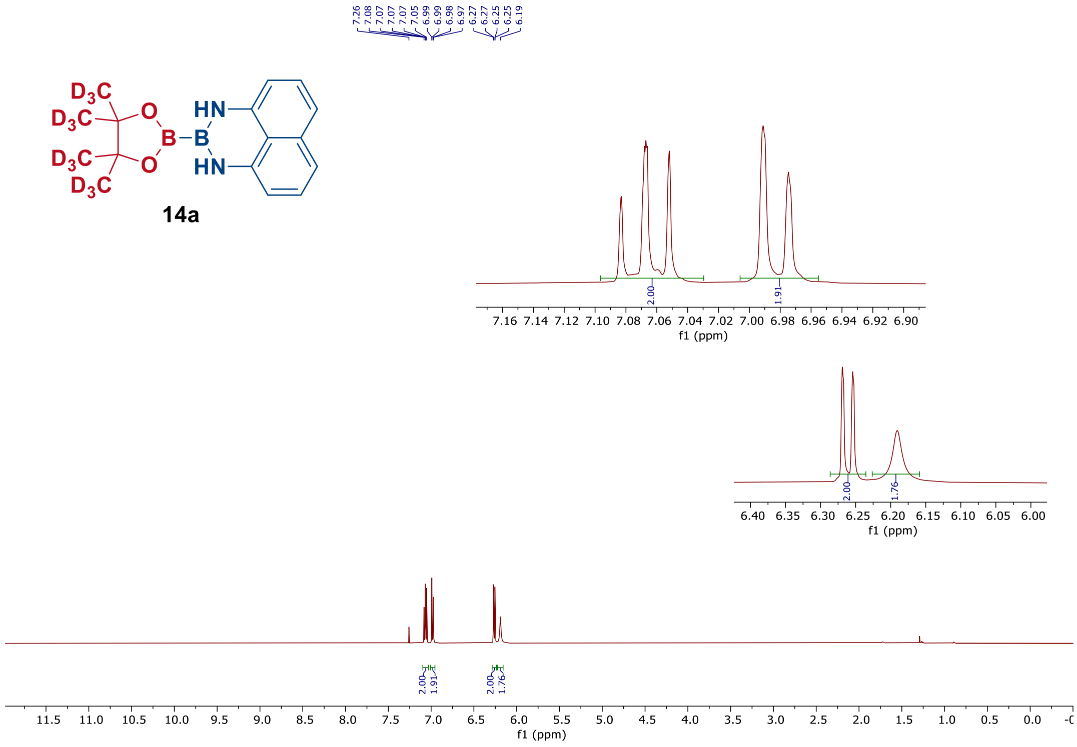

^{1}H NMR (400 MHz, $CDCl_3$) of compound (**14a**).

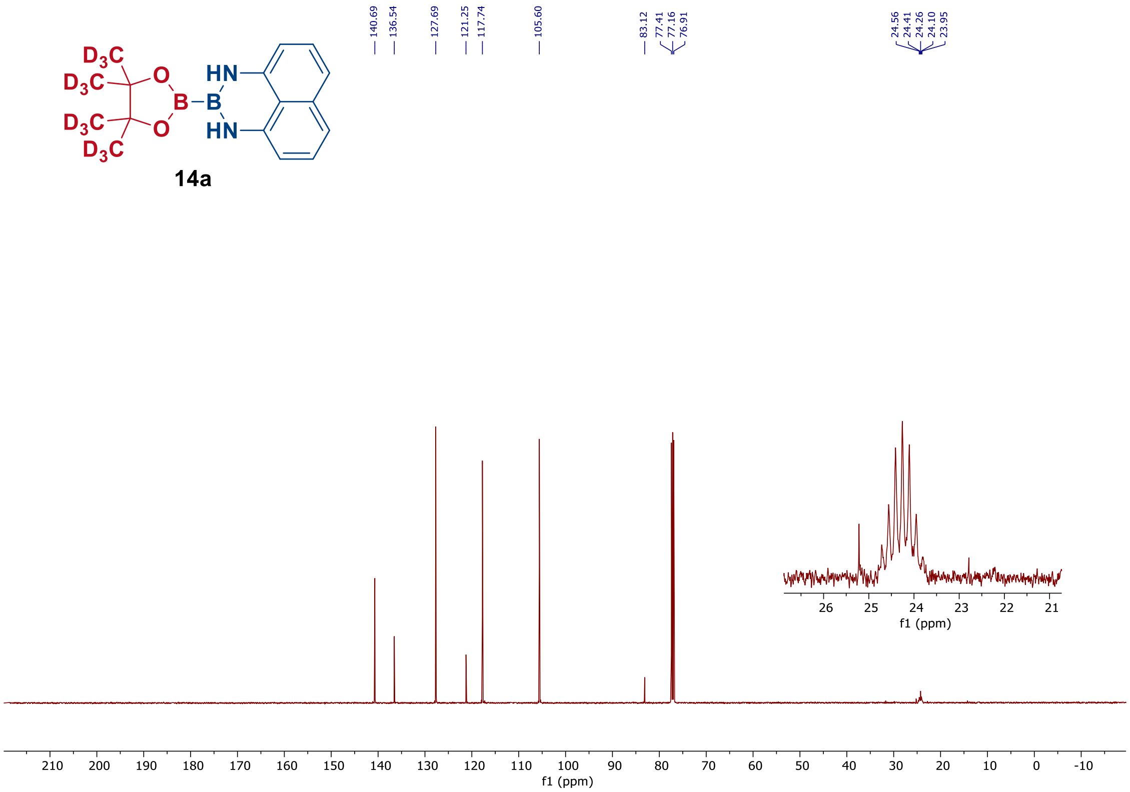

$^{13}C\{^{1}H\}$ NMR (101 MHz, $CDCl_3$) of compound (**14a**).

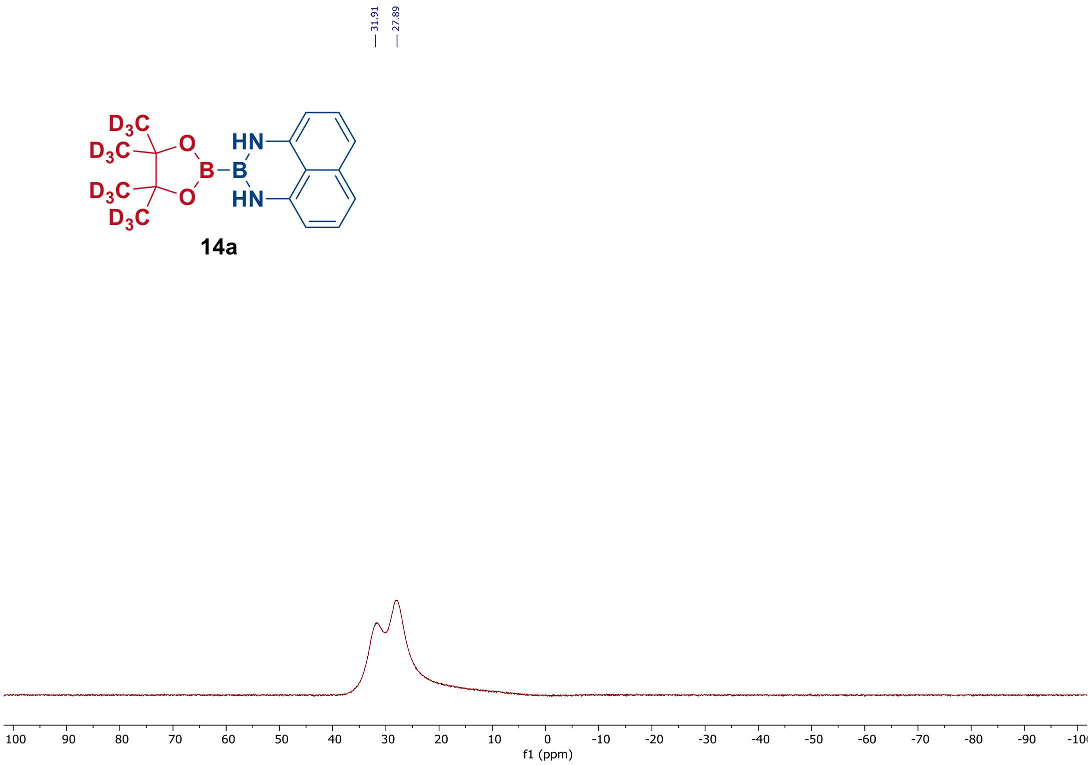

^{11}B NMR (128 MHz, $CDCl_3$) of compound (**14a**).

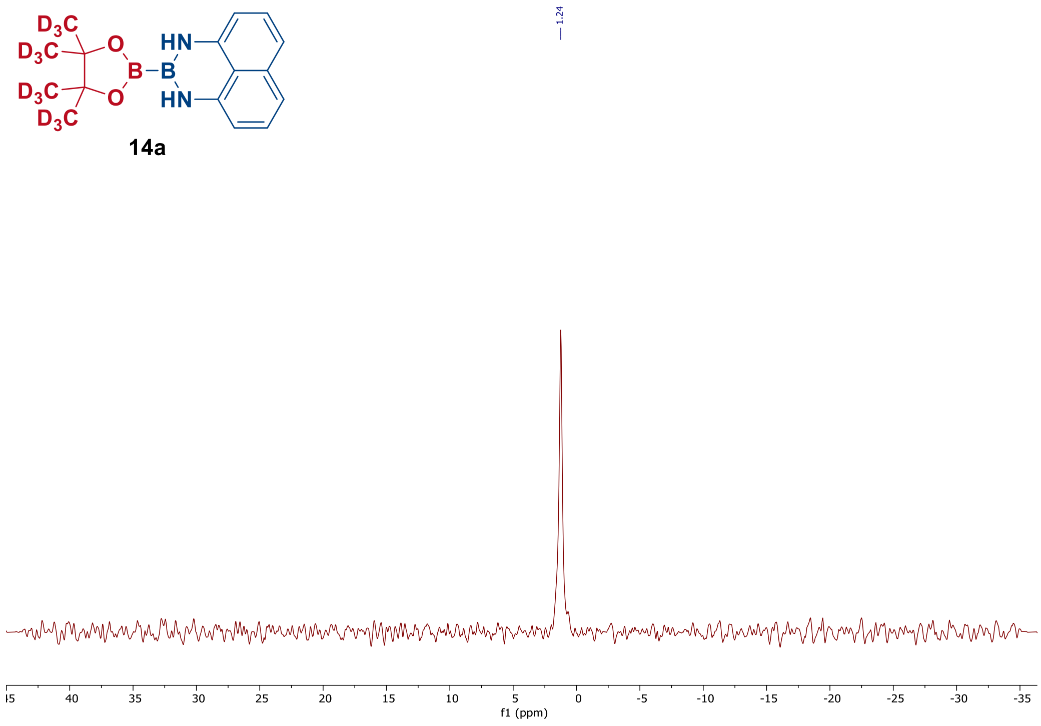

^{2}H NMR (77 MHz, $CHCl_3$) of compound (**14a**).

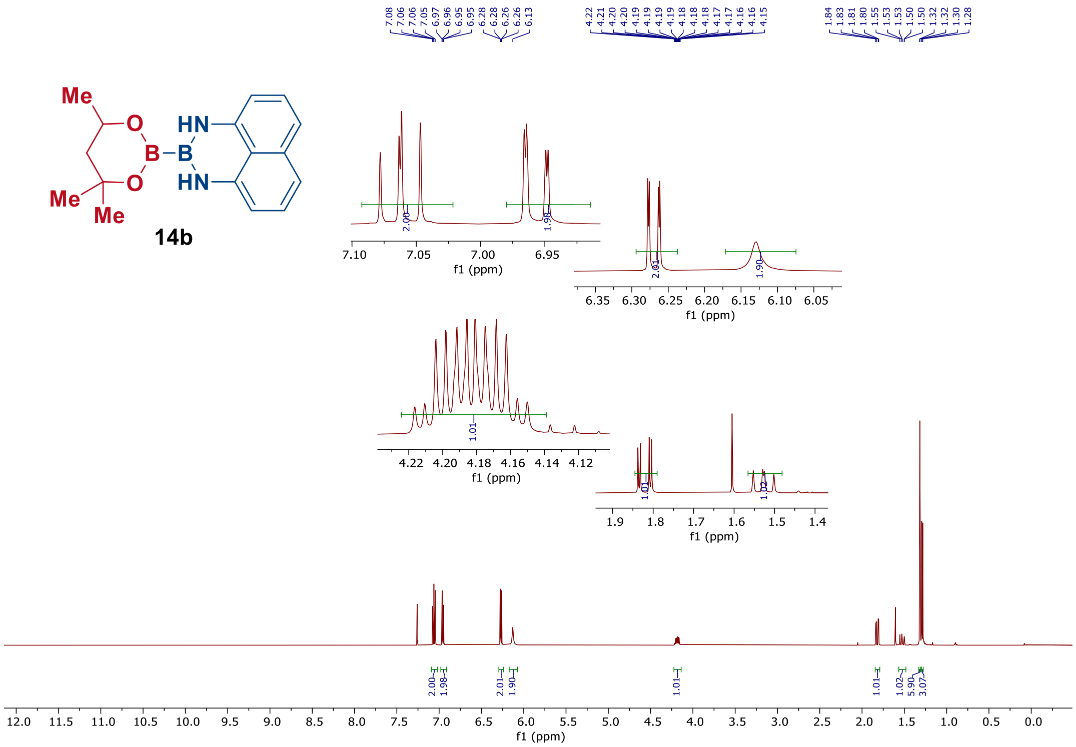

^{1}H NMR (400 MHz, $CDCl_3$) of compound (**14b**).

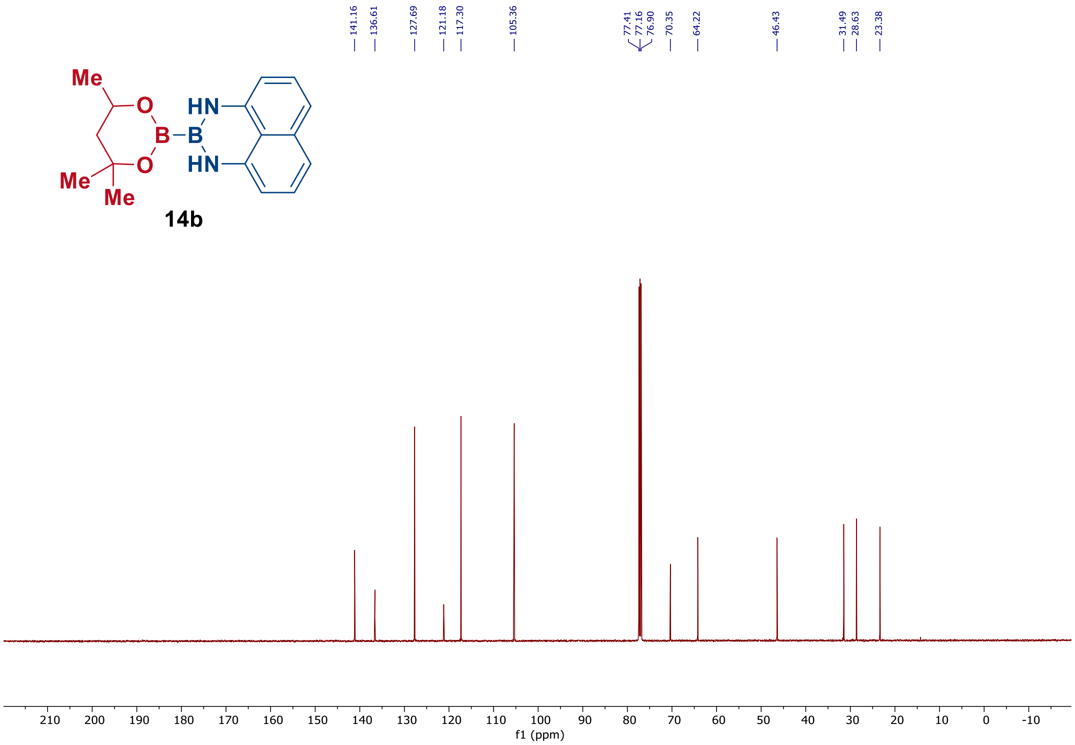

$^{13}C\{^{1}H\}$ NMR (101 MHz, $CDCl_3$) of compound (**14b**).

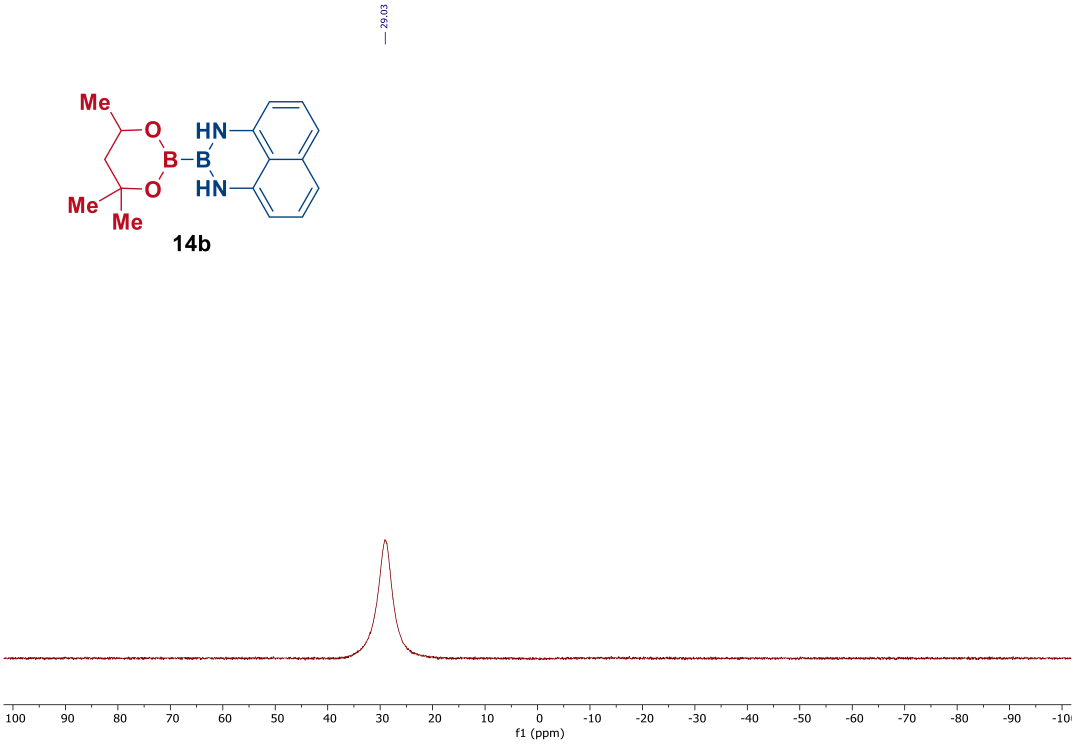

^{11}B NMR (128 MHz, $CDCl_3$) of compound (**14b**).

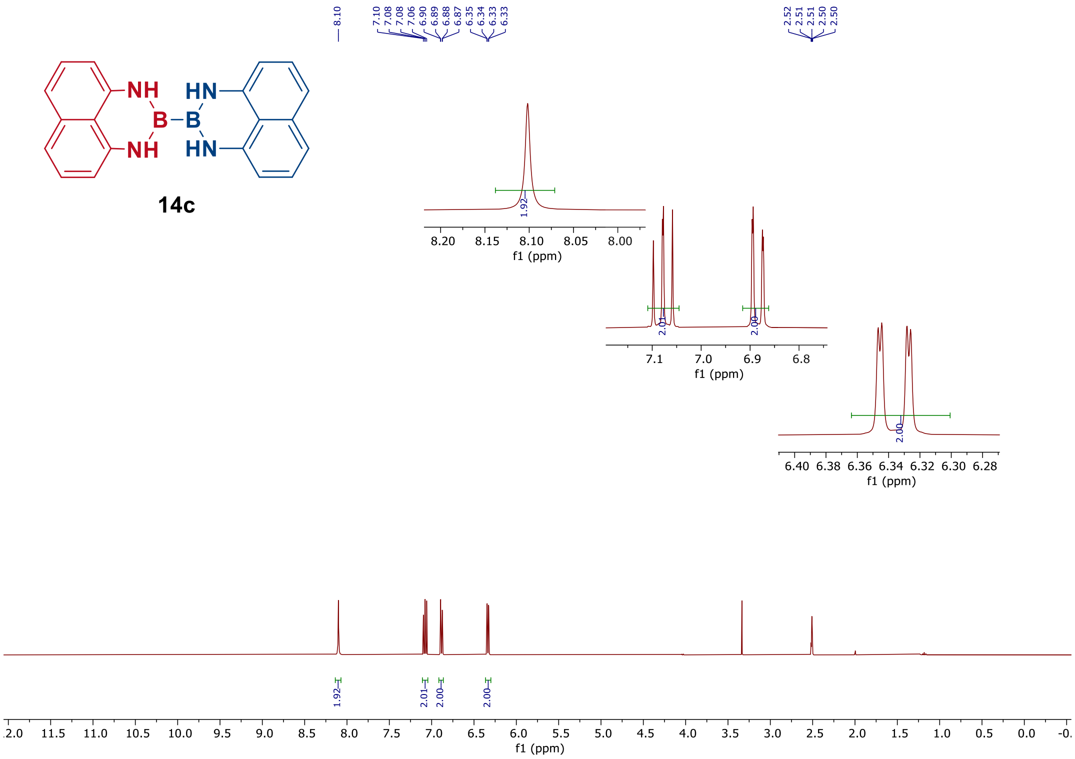

^{1}H NMR (400 MHz, DMSO-*d6*) of compound (**14c**).

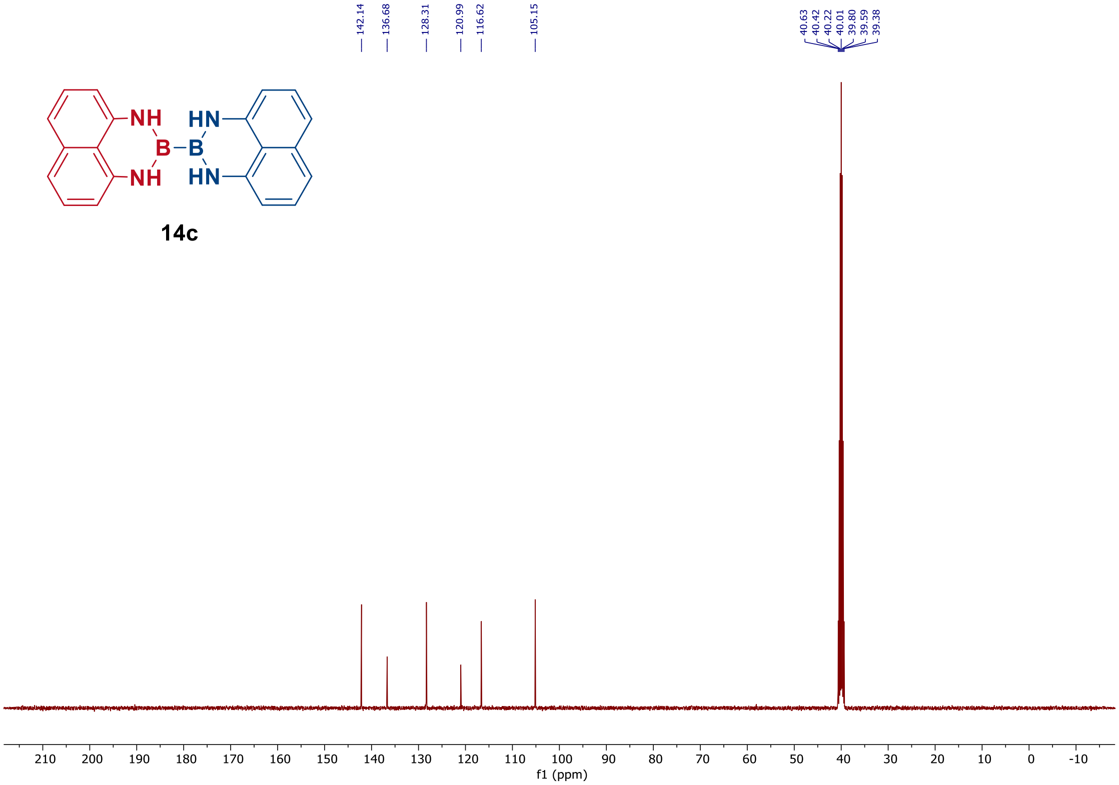

$^{13}C\{^{1}H\}$ NMR (101 MHz, DMSO-*d6*) of compound (**14c**).

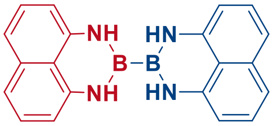

14c

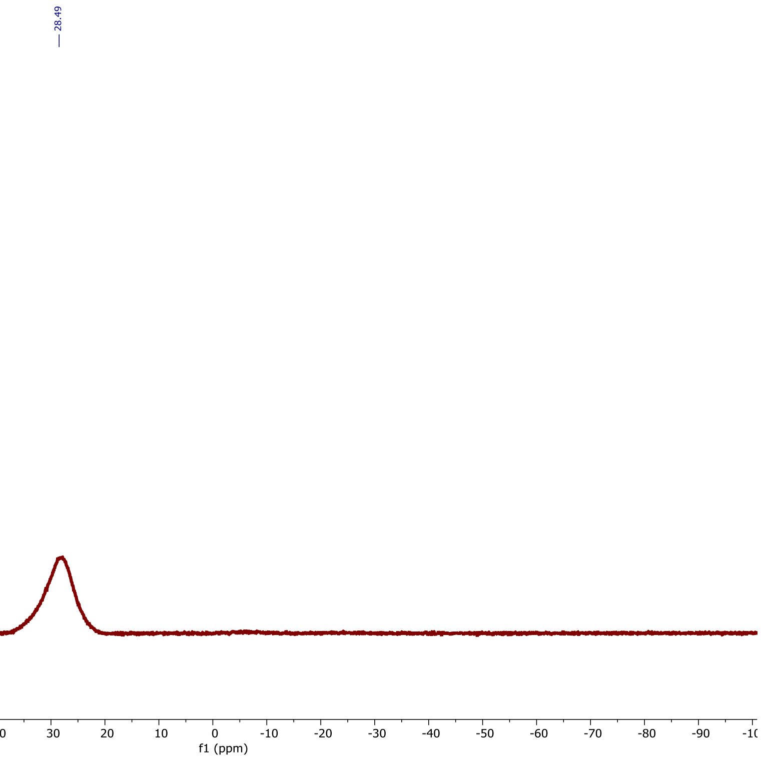

^{11}B NMR (128 MHz, DMSO-*d6*) of compound (**14c**).

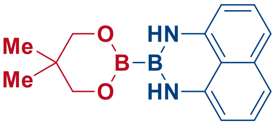

14d

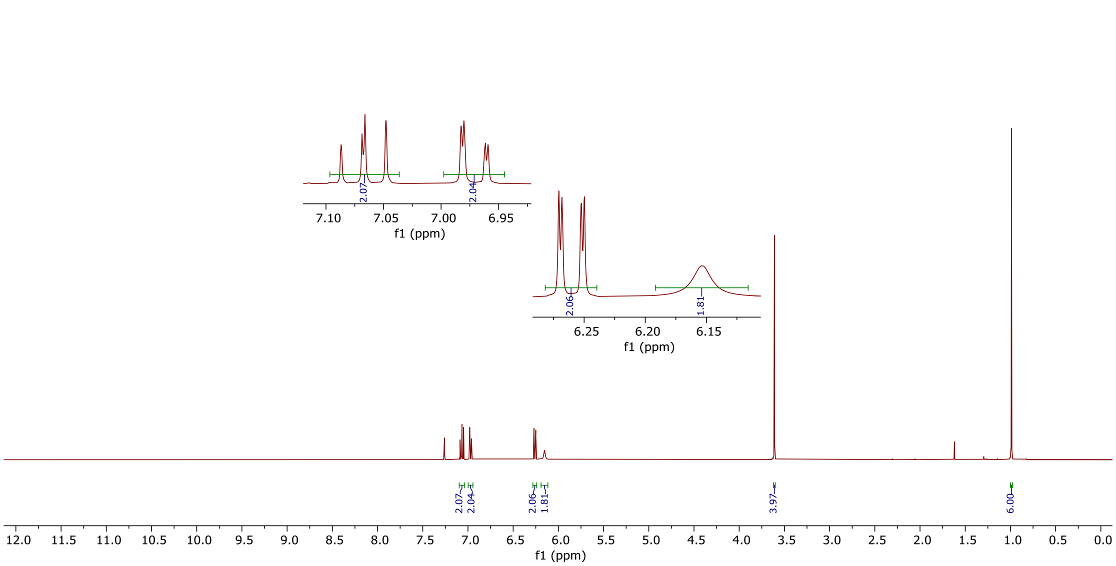

^{1}H NMR (400 MHz, $CDCl_3$) of compound (**14d**).

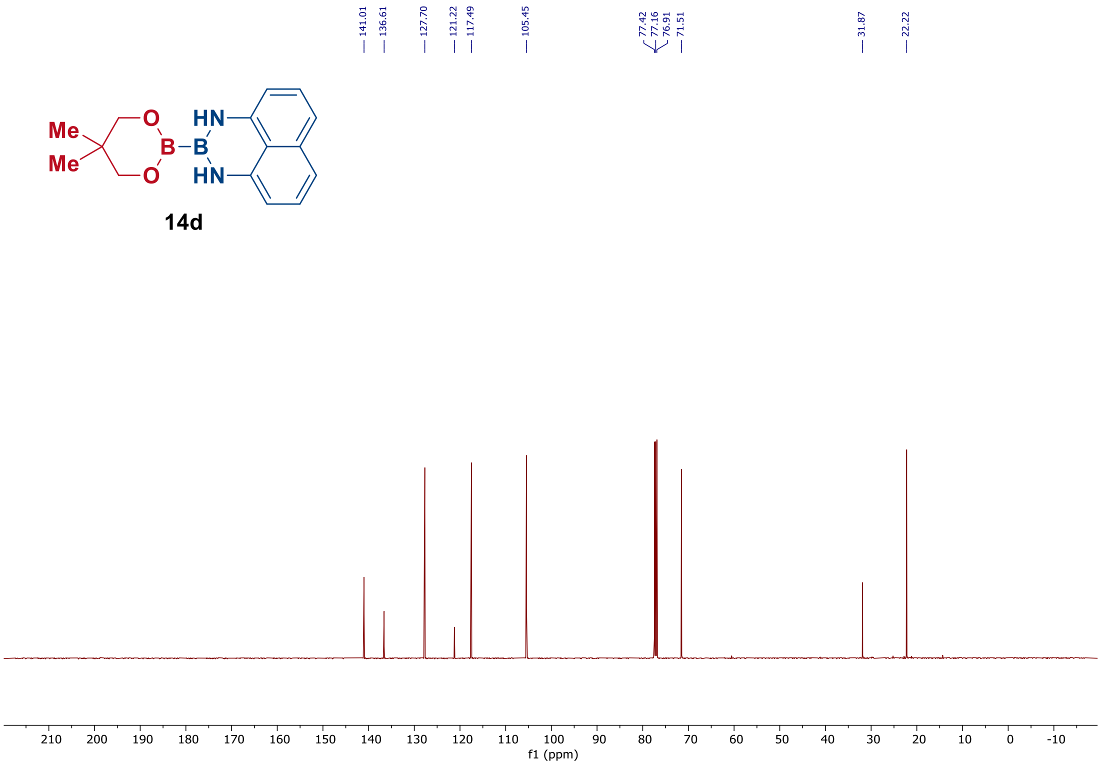

$^{13}C\{^{1}H\}$ NMR (101 MHz, $CDCl_3$) of compound (**14d**).

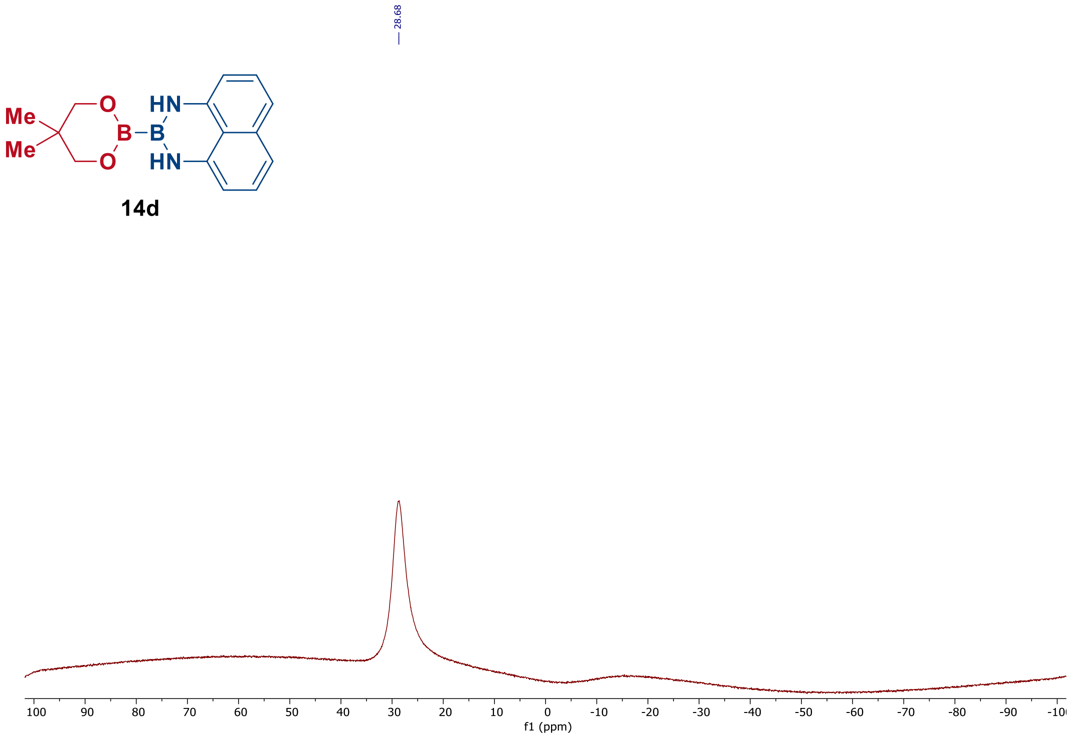

^{11}B NMR (128 MHz, $CDCl_3$) of compound (**14d**).

5. References.

1- Spek, A. L. Single-crystal structure validation with the program PLATON. *J. Appl. Crystallogr*. **2003**, *36*, 7–13.

2- Li, X.; Yuan, L.; Wang, Q.; Liang, L.; Huang, G.; Li, X.; Zhang, C.; Liu, S.; Liu, J. Solid-phase synthesis for novel nerve agent adducted nonapeptides as biomarkers. *Tetrahedron. Letters.* **2017**, *58*, 1437–1440.

3- Yoshida, H.; Murashige, Y.; Osaka, I. Preparation of (pin)B–B(dan). *Org*. *Synth*. **2018**, *95*, 218−230.

4- Yoshida, H.; Michinari, S.; Kageyuki, I.; Osaka, I.; Hatano, S.; Abe, M. B(MIDA)-Containing Diborons. *ACS Omega* **2017**, *2*, 5911–5916.

5- Yasuda, T.; Yoshigoe, Y.; Saito, S. Copper Catalyzed Borylation of Styrenes by 1,8-Diaminonaphthalene-Protected Diboronic Acid. *Org. Lett*. **2023**, *25*, 2093−2097.

6- Yoshida, H.; Murashige, Y.; Osaka, I. Copper-Catalyzed B(dan)-Installing Allylic Borylation of Allylic Phosphates. *Adv*. *Synth*. *Catal*. **2019**, *361*, 2286–2290.

7- Dolomanov, O. V.; Bourhis, L. J.; Gildea, R. J; Howard, J. A. K.; Puschmann, H. OLEX2: A Complete Structure Solution, Refinement and Analysis Program. *Journal of Applied Crystallography*, **2009**, *42*, 339–341.

8- Sheldrick, G. M. Crystal Structure Refinement with *SHELXL*. *Acta Cryst*, **2015**, *A71*, 3-8.

9- Sheldrick, G. M. Crystal Structure Refinement with *SHELXL*. *Acta Cryst*, **2015**, *C71*, 3-8.
